# Supplementary material for: Molecular Basis for Autosomal-Dominant Renal Fanconi Syndrome Caused by HNF4A
Source: Cell Rep. 2019 Dec 24;29(13):4407–4421.e5. doi: 10.1016/j.celrep.2019.11.066 (PMC6941224; doi:10.1016/j.celrep.2019.11.066)
Supplement: Document S1. Figures S1–S7 and Tables S1–S3 [file mmc1.pdf]

Cell Reports, Volume *29*

Supplemental Information

Molecular Basis for Autosomal-Dominant Renal Fanconi Syndrome Caused by *HNF4A*

Valentina Marchesin, Albert Pérez-Martí, Gwenn Le Meur, Roman Pichler, Kelli Grand, Enriko D. Klootwijk, Anne Kesselheim, Robert Kleta, Soeren Lienkamp, and Matias Simons

Table S1: Table summarizing the dominant negative effects (genomic and non-genomic) observed in all the different genotypes. Related to Figure 6.

	Genomic effect			Non-genomic effect			
**PHENOTYPE / GENOTYPE**	**Lipid droplets**	**Mito-chondria ATP5A levels**	**Nuclear localization**	**Autophagy**	**ER**	**Animal viability (with *Dot*-GAL4)**	**Nephrocyte Viability (with *Sns*-GAL4)**
**dHNF4 RNAi**	High	Low	/	Normal	Normal	Normal	Normal (not shown)
**dHNF4^{lowOE} (18°C)**	Very low	Normal (data not shown)	Normal	/	Normal	Normal	Normal (not shown)
**dHNF4^{OE} (25°C)**	Dual pheno-type	High (LD-) Normal (LD+)	Normal (LD-) Peripheral (LD+)	High	Normal (LD-) Expanded (LD+)	Normal (loss of NPs in adults)	Reduced size and number
**dHNF4^{highOE} (29°C)**	Very high	Low	Very peripheral or absent	High (not shown)	Very expanded	Early lethality	/
**dHNF4^{S169A} (25°C/29°C)**	Very low	Very high	Normal	Normal	Normal	Normal	Normal (not shown)
**dHNF4$^{R171W/MODY}$ (25°C)**	Low	High	Normal	Normal	Normal	Normal	Normal
**dHNF4$^{R171W/MODY}$ (29°C)**	Low	/	Normal	/	Normal	Normal	/
**dHNF4$^{R167W/FRTS}$ (25°C)**	Very high	Low	Very peripheral or absent	High	Very expanded	Early lethality	Loss of NPs
**dHNF4$^{R167W/FRTS}$ (18°C)**	Very high	Low (not shown)	Very peripheral or absent	/	Very expanded	Early lethality	/
**dHNF4$^{R167W/S169A}$ (25°C)**	High (partial rescue)	Normal	Normal	Normal	Normal	Normal	Normal

/: data not available

Table S2. ***Drosophila*** **genotypes used in this study. Related to STAR Methods.**

Genotype	Figure
w; +/+; *hnf4*-HNF4-GFP.Flag	Figure 1
w; +/+; *hsp70*-GAL4-dHNF4, UAS-nlacZ56	Figure 1
w; *Dot*-Gal4/+; UAS-*GFP-RNAi* (bloo#9330)/+	Figures 1, 2, 4, 6, S1, S2, S3, S4, S5, S6
w; *Dot*-Gal4/UAS-*Pkd2-RNAi* (bloo#51502); +/+	Figure S1
w/yv; *Dot*-Gal4/+; UAS-*HNF4-RNAi* (bloo#29375)/+	Figures 1, S1, S4
w; *Dot*-Gal4/UAS-*HNF4-RNAi* (Vienna GD12692); +/+	Figure S1
w; *Dot*-Gal4/+; UAS-HNF4-3xHA/+ (FlyORF #F000144)	Figures 2, 3, S2, S3, S4
w; *Dot*-Gal4/UAS-*midway-RNAi* (bloo#65963); +/+	Figure S2
w/yv; *Dot*-Gal4/+; UAS-*GFP-RNAi*/UAS-*HNF4-RNAi* (bloo#29375)	Figure S2
w/yv; *Dot*-Gal4/UAS- *midway-RNAi*; UAS-*HNF4-RNAi* (bloo#29375)/+	Figure S2
w; *Dot*-Gal4/UAS-*midway-RNAi*; UAS-HNF4-3xHA/+	Figure S2
w; *Dot*-Gal4/+; UAS-HNF4^{R167W}-3xHA/+	Figures 5, 6, S6
w; *Dot*-Gal4/+; UAS-HNF4^{R171W}-3xHA/+	Figures 5, 6, S6
w; *Dot*-Gal4/+; UAS-HNF4^{S169A}-3xHA/+	Figures 4, S5
w; *Dot*-Gal4/+; UAS-HNF4$^{R167W/S169A}$-3xHA/+	Figures 5, 6
w; *Dot*-Gal4/ UAS-*midway-RNAi*; UAS-HNF4^{R167W}-3xHA/+	Figure S6
w; *Dot-Gal4*/UAS-*Pkd2-RNAi*; *hnf4*-HNF4-GFP.Flag/+	Figure 4
w; *Dot-Gal4*/+; *hnf4*-HNF4-GFP.Flag/UAS-HNF4-3xHA	Figure 4
w; *Dot-Gal4*/+; *hnf4*-HNF4-GFP.Flag/UAS-HNF4^{R167W}-3xHA	Figure 5
w; *Dot-Gal4*/+; *hnf4*-HNF4-GFP.Flag/UAS-HNF4^{R171W}-3xHA	Figure 5
w; *Dot-Gal4*/+; *hnf4*-HNF4-GFP.Flag/UAS-HNF4^{S169A}-3xHA	Figure 4
w; *Dot-Gal4*/+; *hnf4*-HNF4-GFP.Flag/UAS-HNF4$^{R167W/S169A}$-3xHA	Figure 5
Dot-Gal4; UAS-GFP-mCherry-Atg8/UAS-*Pkd2-RNAi*; +/+	Figures S4, S5, S6
Dot-Gal4; UAS-GFP-mCherry-Atg8/UAS-*ATP6AP2-RNAi* (Vienna KK105281); +/+	Figure S4
w; *Dot-Gal4/*UAS-GFP-mCherry-Atg8; UAS-HNF4-3XHA/+	Figure S4
w; *Dot-Gal4/*UAS-GFP-mCherry-Atg8; UAS-HNF4^{R167W}-3XHA/+	Figure S6

Dot-Gal4; UAS-GFP-mCherry-Atg8/+; UAS-HNF4^{R171W}-3XHA/+	Figure S6
w; *Dot-Gal4/*UAS-GFP-mCherry-Atg8; UAS-HNF4$^{R167W/S169A}$-3XHA/+	Figure S6
Dot-Gal4; UAS-GFP-mCherry-Atg8/+; UAS-HNF4^{S169A}-3XHA/+	Figure S6
w; *Sns*-GAL4/+; UAS-*GFP-RNAi*/+	Figures 6, S1, S4
w; *Sns*-GAL4/+; UAS-*HNF4-RNAi* (bloo#29375)/+	Figure S1
w; *Sns*-GAL4/+; UAS-HNF4-3xHA/+	Figure S4
w; *Sns*-GAL4/+; UAS-HNF4^{R167W}-3xHA/+	Figure 6
w; *Sns*-GAL4/+; UAS-HNF4^{R171W}-3xHA/+	Figure 6
w; *Sns*-GAL4/+; UAS-HNF4$^{S169A/R167W}$-3xHA/+	Figure 6

Table S3. Oligonucleotide information. Related to STAR Methods.

Samples	Oligonucleotides
Mutagenesis in flies: UAS-HNF4^{R167W}	forward:CATTCTTCTGGAGGAGTGTCAGGAAAAATCATCAG<br>reverse:ACTCCTCCAGAAGAATCCTTTGCAGCCGTCGCAGC
Mutagenesis in flies: UAS-HNF4^{S169A}	forward:CAGGAGGGCTGTCAGGAAAAATCATCAGTACAC<br>reverse:CCTGACAGCCCTCCTGAAGAATCCTTTGCAGC
Mutagenesis in flies: UAS-HNF4$^{R167W/S169A}$	forward:CTGGAGGGCTGTCAGGAAAAATCATCAGTACAC<br>reverse:CCTGACAGCCCTCCAGAAGAATCCTTTGCAGC
Mutagenesis in flies: UAS-HNF4^{R171W}	forward:GGAGTGTCTGGAAAAATCATCAGTACACTTGCAG<br>reverse:GGAGTGTCTGGAAAAATCATCAGTACACTTGCAG
Mutagenesis in COS-7 cells: R85W	forward:CGCACGCTCCTCCAGAAGAAGCCCTTG<br>reverse:CAAGGGCTTCTTCTGGAGGAGCGTGCG
Mutagenesis in COS-7 cells: R89W	forward:CATGTGGTTCTTCCACACGCTCCTCCGGA<br>reverse:TCCGGAGGAGCGTGTGGAAGAACCACATG
Mutagenesis in COS-7 cells: S87A	forward:GGTTCTTCCGCACGGCCCTCCGGAAGAAGC<br>reverse:GCTTCTTCCGGAGGGCCGTGCGGAAGAACC
Mutagenesis in COS-7 cells: R85W/S87A	forward:GGTTCTTCCGCACGGCCCTCCAGAAGAAGCCCTTGC<br>reverse:GCAAGGGCTTCTTCTGGAGGGCCGTGCGGAAGAACC
Mutagenesis on human HNF4A: R85W	forward:GGGCTTCTTCTGGAGGAGCGT<br>reverse:GTCGACACTGCCGACGTT
qPCR primers: Tbp	forward:CCCCTTGTACCCTTCACCAAT<br>reverse:GAAGCTGCGGTACAATTCCAG
qPCR primers: HNF4A	forward:GGTGTCCATACGCATCCTTGAC<br>reverse:AGCCGCTTGATCTTCCCTGGAT
qPCR primers: V5.HNF4A	forward:GTAAGCCTATCCCTAACCCTCTC<br>reverse:AAATTCCAGGGTGGTGTAGG
