# Supplementary material for: Molecular Basis for Autosomal-Dominant Renal Fanconi Syndrome Caused by HNF4A
Source: Cell Rep. 2019 Dec 24;29(13):4407–4421.e5. doi: 10.1016/j.celrep.2019.11.066 (PMC6941224; doi:10.1016/j.celrep.2019.11.066)
Supplement: Document S2. Article plus Supplemental Information [file mmc2.pdf]

Cell Reports

Article

Molecular Basis for Autosomal-Dominant Renal Fanconi Syndrome Caused by *HNF4A*

Graphical Abstract

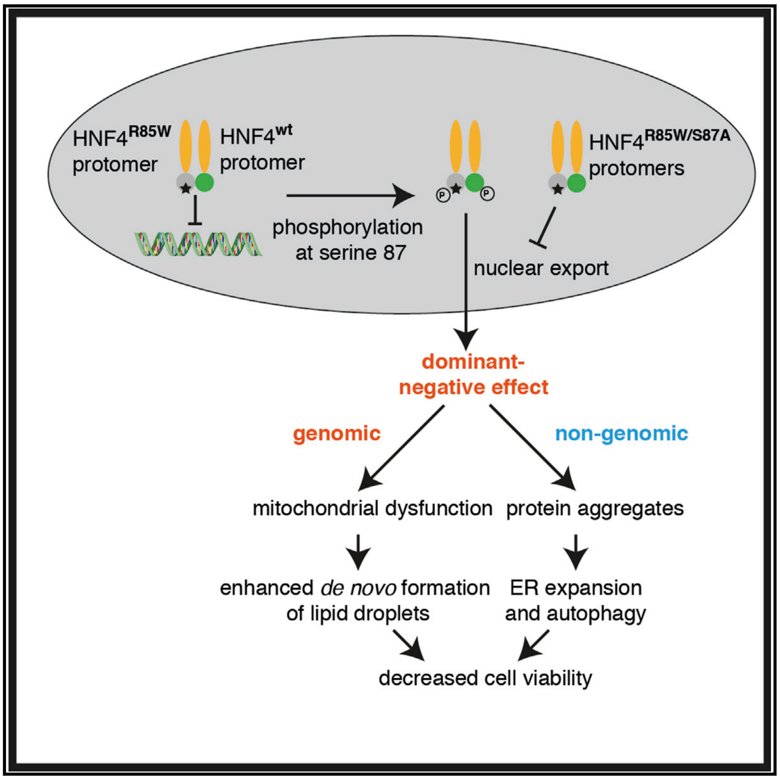

Authors

Valentina Marchesin, Albert Pérez-Martí, Gwenn Le Meur, ..., Robert Kleta, Soeren Lienkamp, Matias Simons

Correspondence

matias.simons@institutimagine.org

In Brief

Mutations in HNF4A cause the autosomal-dominant maturity-onset diabetes of the young type 1 (MODY1). One particular mutation in the DNA-binding domain, p.R85W, causes additional kidney defects. Using *Drosophila* nephrocytes as a model, Marchesin et al. show that this mutation causes lipid metabolism defects and mitochondrial dysfunction in a dominant-negative manner.

Highlights

- HNF4 controls lipid metabolism in *Drosophila* nephrocytes
- The kidney disease mutation R85W shows dominant-negative effects in nephrocytes
- Dephosphorylation at S87 prevents the dominant-negative effects
- R85W mutation causes mitochondrial dysfunction in reprogrammed renal epithelial cells

Marchesin et al., 2019, Cell Reports *29*, 4407–4421
December 24, 2019 © 2019 The Authors.
https://doi.org/10.1016/j.celrep.2019.11.066

Cell Reports
Article

OPEN ACCESS
CellPress

Molecular Basis for Autosomal-Dominant Renal Fanconi Syndrome Caused by *HNF4A*

Valentina Marchesin,[1] Albert Pérez-Martí,[1] Gwenn Le Meur,[1] Roman Pichler,[2] Kelli Grand,[3] Enriko D. Klootwijk,[4] Anne Kesselheim,[4] Robert Kleta,[4] Soeren Lienkamp,[2,3] and Matias Simons[1,5,*]
[1]INSERM UMR1163, Laboratory of Epithelial Biology and Disease, Imagine Institute, Paris Descartes University, Sorbonne Paris Cité, Hôpital Necker-Enfants Malades, 75015 Paris, France
[2]Renal Division, University Medical Center Freiburg, Faculty of Medicine, University of Freiburg, 79098 Freiburg, Germany
[3]Institute of Anatomy, University of Zurich, 8057 Zurich, Switzerland
[4]Department of Renal Medicine, University College London, London NW3 2PF, UK
[5]Lead Contact
*Correspondence: matias.simons@institutimagine.org
https://doi.org/10.1016/j.celrep.2019.11.066

SUMMARY

HNF4A is a nuclear hormone receptor that binds DNA as an obligate homodimer. While all known human heterozygous mutations are associated with the autosomal-dominant diabetes form MODY1, one particular mutation (p.R85W) in the DNA-binding domain (DBD) causes additional renal Fanconi syndrome (FRTS). Here, we find that expression of the conserved fly ortholog dHNF4 harboring the FRTS mutation in *Drosophila* nephrocytes caused nuclear depletion and cytosolic aggregation of a wild-type dHNF4 reporter protein. While the nuclear depletion led to mitochondrial defects and lipid droplet accumulation, the cytosolic aggregates triggered the expansion of the endoplasmic reticulum (ER), autophagy, and eventually cell death. The latter effects could be fully rescued by preventing nuclear export through interfering with serine phosphorylation in the DBD. Our data describe a genomic and a non-genomic mechanism for FRTS in HNF4A-associated MODY1 with important implications for the renal proximal tubule and the regulation of other nuclear hormone receptors.

INTRODUCTION

HNF4A (HNF4α, NR2A1) is a transcription factor that belongs to the nuclear hormone receptor superfamily. HNF4A plays a critical role in development, cell differentiation, and metabolism, particularly in the visceral endoderm, liver, intestine, kidney, and pancreatic β cells. The most important target genes of HNF4A belong to glucose, lipid, and drug metabolism pathways (Cattin et al., 2009; Fang et al., 2012; Maestro et al., 2007). Like all other nuclear hormone receptors, HNF4A has a ligand-binding domain and a DNA-binding domain (LBD and DBD, respectively). Long considered an orphan receptor, the LBD of HNF4A binds fatty acids in a reversible fashion, as suggested by several reports (Duda et al., 2004; Wisely et al., 2002). The highly conserved DBD recognizes the bipartite direct repeat 1 (DR1) consensus sequence that is present in a great variety of target genes (Fang et al., 2012). Although HNF4A only dimerizes with itself and not with other nuclear receptors (Jiang et al., 1995), it was recently shown that an increase in functional diversity can be achieved by the dimerization of different splice variants (Ko et al., 2019). The levels of nuclear HNF4A are regulated by protein kinase C (PKC)-dependent phosphorylation of a serine residue at position 87 in the DNA-binding domain, leading to enhanced nuclear export and proteasomal degradation (Sun et al., 2007).

More than 2 decades ago, mutations in the *HNF4A* gene were identified as the first monogenic cause of maturity-onset diabetes of the young type 1 (MODY1) (Yamagata et al., 1996), usually diagnosed before the age of 25 years in patients with negative islet cell autoantibodies. Additional MODY genes are *HNF1A* and *HNF1B*, which functionally interact with HNF4A, thereby defining a transcriptional network responsible for phenotypically indistinguishable forms of MODY (Duncan et al., 1998). Thus far, ~100 MODY1 mutations have been described for the coding region of *HNF4A* (Anık et al., 2015). All of them are dominant mutations, considered to be haploinsufficient (Ferrer, 2002). One specific missense mutation in the DNA-binding domain, p.R85W, causes additional defects in the proximal tubules of the kidney or Fanconi renotubular syndrome (FRTS; OMIM: 616026; note that due to differences in genome annotations, this mutation has also been called R76W and R63W). This mutation has been reported in 15 cases from 12 unrelated families with full penetrance of the FRTS in the carriers (Clemente et al., 2017; Hamilton et al., 2014; Improda et al., 2016; Liu et al., 2018; Numakura et al., 2015; Stanescu et al., 2012; Walsh et al., 2017). In the kidney, HNF4A is exclusively expressed in proximal tubules (Marable et al., 2018). However, why only the R85W mutation in HNF4A causes FRTS remains unclear.

The proximal tubule is situated close to the glomerulus and is required for the extensive reabsorption of water, electrolytes, and specific organic solutes. This includes low-molecular-weight (LMW) proteins, protein-bound lipids, amino acids, glucose, bicarbonate, and phosphate. Uptake occurs via receptor-mediated endocytosis or transepithelial transport, which requires an unusually high amount of energy in the

This is an open access article under the CC BY license (http://creativecommons.org/licenses/by/4.0/).

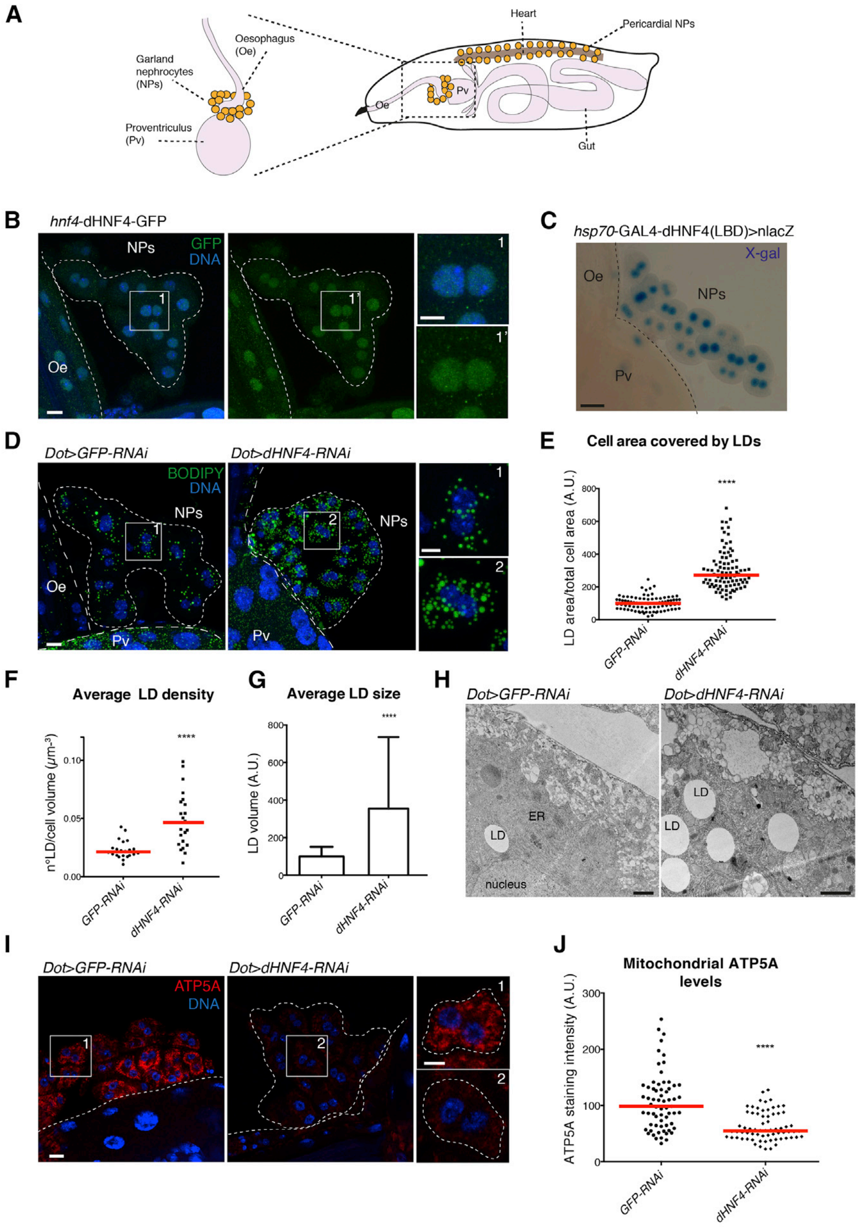

Figure 1. dHNF4 Silencing Increases Lipid Droplet Content and Causes Mitochondrial Dysfunction in *Drosophila* Nephrocytes

(A) In *Drosophila* third instar larvae, garland nephrocytes (NPs) are tethered to the esophagus (Oe) and proventriculus (Pv) and pericardial nephrocytes are tethered to the heart.

(B) Confocal section with insets showing garland nephrocytes expressing dHNF4-GFP under the native *dHNF4* promoter, stained for GFP and DNA. Scale bars, 10 μm, insets 5 μm.

(C) Bright-field images of garland cells expressing a reporter for dHNF4 activation, stained for lacZ expression with X-gal. Scale bars, 10 μm.

(D) Z-projections showing staining with BODIPY 493/503 for lipid droplets in nephrocytes expressing RNAi against *GFP* and *dHNF4*. Scale bars,10 μm, insets 5 μm.

(E) Quantification of the cell area occupied by LDs per cell. Values were normalized to the average value in control nephrocytes. Scattered plot represents seven independent experiments and red lines represent medians. Mann-Whitney t test: ****p < 0.0001.

(F) Quantification of the average LD density measured by the number of LDs per cell volume (two independent experiments, red lines: medians, Mann-Whitney t test: ****p < 0.0001).

(G) Quantification of the average LD size. Values were normalized to the average value in control nephrocytes, and bars represent means ± SDs (two independent experiments, Mann-Whitney t test: ****p < 0.0001).

(H) TEM sections of GFP and dHNF4-depleted nephrocytes. Scale bars, 1 μm.

(I) Confocal sections of GFP and dHNF4-depleted nephrocytes stained for ATP5A and DNA. Scale bars, 10 μm, insets 5 μm.

(J) Quantification of the ATP5A staining intensity. Values were normalized to the average value in control nephrocytes (four independent experiments, red lines: medians, Mann-Whitney t test: ****p < 0.0001).

See also Figures S1 and S2.

form of ATP. In proximal tubule cells, ATP is for the most part generated by mitochondrial β-oxidation of fatty acids (Kang et al., 2015), which are mostly taken up from the environment (Bobulescu, 2010). FRTS, a failure of proximal tubular function, is therefore featured by LMW proteinuria, glucosuria, aminoaciduria, and phosphaturia, with some forms leading to end-stage renal disease. The most common are genetic forms affecting endolysosomal or mitochondrial functions (Klootwijk et al., 2015).

In *Drosophila*, HNF4A is well conserved (dHNF4), and its inactivation causes the accumulation of lipid droplets (LDs) in various tissues due to reduced mitochondrial function (Bülow et al., 2018; Palanker et al., 2009). Here, we used *Drosophila* nephrocytes as a model for proximal tubules to study the function of HNF4A and the impact of the FRTS-associated R85W mutation. These cells are specialized in the reabsorption of components from the hemolymph (Helmstädter and Simons, 2017; Weavers et al., 2009). The reabsorption includes filtration via slit diaphragms and then, similar to proximal tubular cells, endocytic uptake via the LMW receptors cubilin and amnionless (Helmstädter and Simons, 2017; Zhang et al., 2013a). We found that that the expression of dHNF4 harboring the FRTS mutation affects the catabolism of LDs in nephrocytes by interfering with mitochondrial function, which could be confirmed in a mammalian renal epithelial system (Kaminski et al., 2016). Moreover, we showed that the FRTS mutation promotes the nuclear export of a wild-type reporter protein, thereby reducing transcriptional output in a dominant-negative manner and promoting the formation of cytotoxic dHNF4 aggregates in the cytosol. The cytotoxic effects could be suppressed by

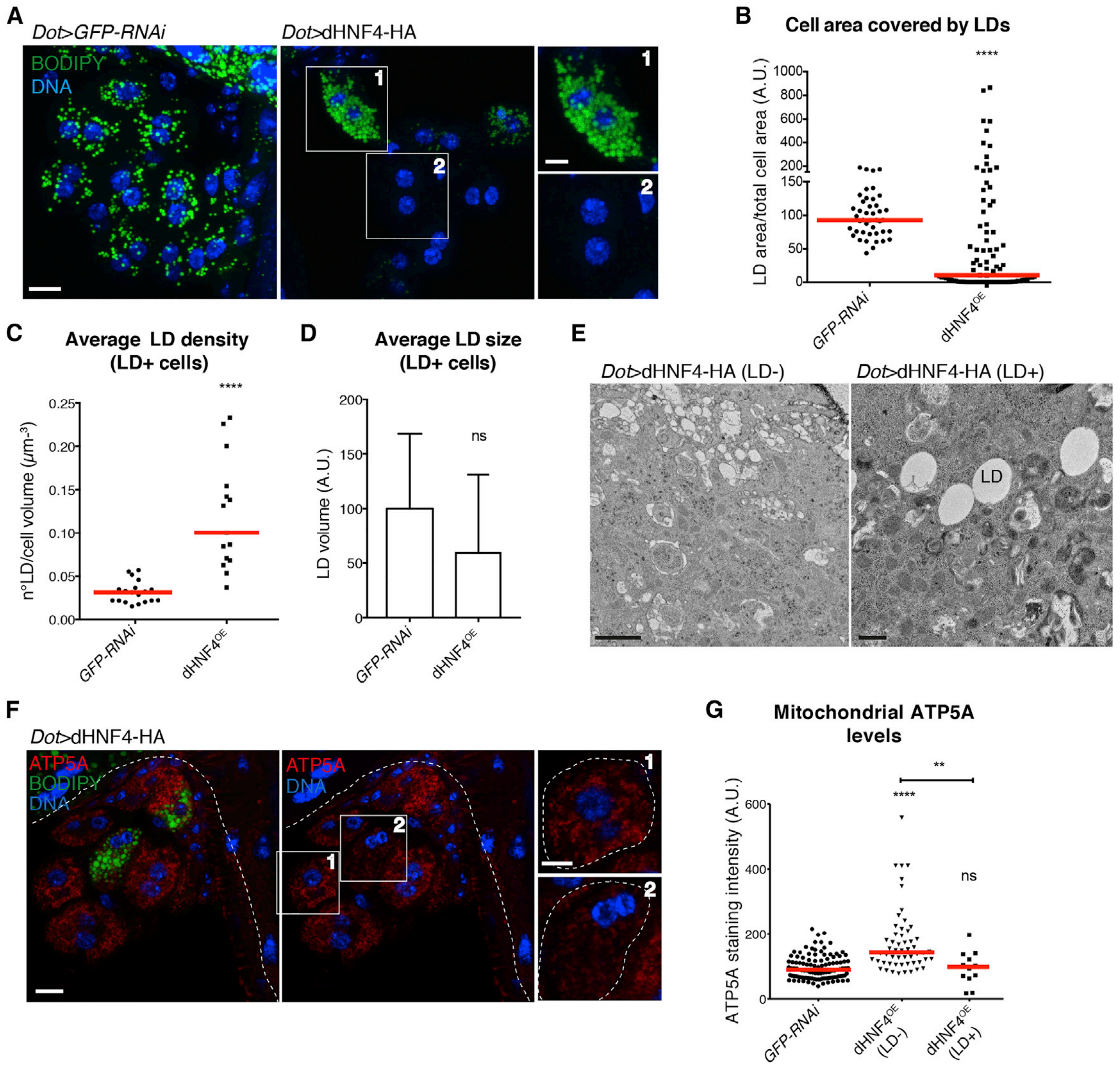

Figure 2. dHNF4 Overexpression Decreases Lipid Droplet Content

(A) Z-projections with insets showing LD and DNA staining in control and dHNF4-overexpressing nephrocytes. Scale bars, 10 µm, insets 5 µm.

(B) Quantification of the cell area covered by LDs per cell. Values were normalized to the average value in control nephrocytes (five independent experiments, red lines: medians, Mann-Whitney t test: ****p < 0.0001).

(C) Quantification of the average LD density (number of LDs per cell volume) in control and dHNF4-overexpressing nephrocytes (three independent experiments, red lines: medians, Mann-Whitney t test: ****p < 0.0001).

(D) Quantification of the average LD size. Values were normalized to the average value in control nephrocytes and bars represent means ± SDs (three independent experiments, red lines: medians, Mann-Whitney t test: non-significant [ns]).

(E) TEM sections of an LD^{-} and an LD^{+} dHNF4-overexpressing nephrocyte. Scale bars, 1 µm.

(F) Confocal sections of dHNF4-overexpressing nephrocytes, stained for LD, ATP5A, and DNA. Scale bars, 10 µm, insets 5 µm.

(G) Quantification of the ATP5A staining intensity in LD^{-} and LD^{+} cells shown in (F). Values were normalized to the average value in control nephrocytes (five independent experiments, red lines: medians, one-way ANOVA test: ****p < 0.0001, **p < 0.01, ns: non-significant).

See also Figures S2 and S3.

inhibiting nuclear exit and proteasomal degradation of dHNF4. By contrast, the expression of another known close mutation in the DBD, R89W (that leads to MODY1 but not to FRTS; Hamilton et al., 2014), did not cause any dominant-negative or cytotoxic effects. Our data shed light on the molecular basis for the unique effects of the FRTS mutation, with implications for lipid metabolism in proximal tubules and for the control of other nuclear hormone receptors.

RESULTS

dHNF4 Controls LD Content and Mitochondria in Nephrocytes

Nephrocytes exist in two functionally equivalent populations, one close to the gut (garland nephrocytes) and one close to the heart (pericardial nephrocytes) (Figure 1A). An advantage of the garland nephrocytes is that they can easily be dissected

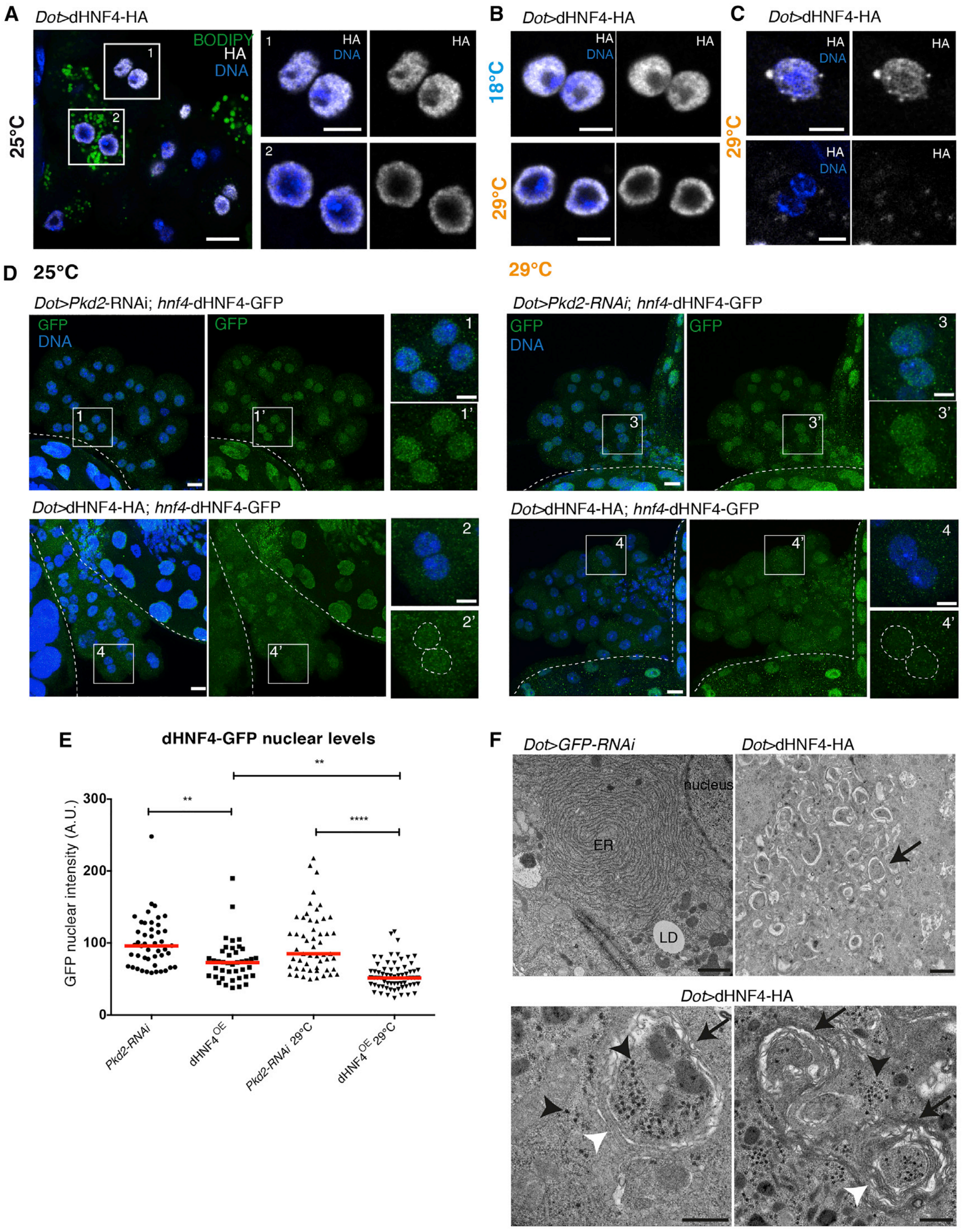

Figure 3. dHNF4highOE Leads to Nuclear Depletion of the Wild-Type Protomer

(A) Confocal sections of nephrocytes overexpressing dHNF4, stained for LD, DNA, and HA, and insets showing nuclei. Scale bars, 10 μm, insets 5 μm.

(B and C) Confocal sections of representative nuclei stained for DNA and HA of nephrocytes overexpressing dHNF4 at 18°C (B) and 29°C (B and C). Scale bars, 5 μm.

(D) Z-projections of nephrocytes expressing dHNF4-GFP under the native *dHNF4* promoter together with control RNAi or dHNF4-HA, at 25°C and 29°C, stained for GFP and DNA. Scale bars, 10 μm, insets 5 μm.

(E) Quantification of the average nuclear GFP intensity in (D). Values were normalized to control nephrocytes at 25°C and 29°C (three independent experiments for each genotype, red bars: medians, one-way ANOVA test: $^{**}p < 0.01$, $^{****}p < 0.0001$).

(legend continued on next page)

and studied *ex vivo.* The expression and active state of dHNF4 in the nuclei of garland nephrocytes was addressed by two different reporter lines: the first expresses GFP-tagged dHNF4 under its native promoter and was shown to rescue the lethality of a *dHNF4* null mutant (Palu and Thummel, 2016); the second reporter line expresses a chimeric protein obtained by the fusion of the DBD of GAL4 and the LBD of dHNF4, which, when activated, drives the expression of a *lacZ* gene (Palanker et al., 2009). While dHNF4-GFP was readily expressed in the nuclei of garland nephrocytes and surrounding gut tissue (Figure 1B), the *lacZ* reporter was active only in nephrocytes, suggesting an important function of dHNF4 in these cells (Figure 1C).

To better understand the function of dHNF4 in nephrocytes, we studied LDs that were previously shown to accumulate in several tissues of starved *dHNF4* null mutants due to a reduction in mitochondrial β-oxidation (Palanker et al., 2009). LDs can be visualized by the lipophilic BODIPY dye that partitions into the triglyceride (triacylglycerol [TAG])- and cholesterylester-filled core of these cytoplasmic organelles. Transgenes were expressed with the UAS-GAL4 system (Brand and Perrimon, 1993) using *Dorothy*-GAL4 (*Dot*-GAL4) and *Sns*-GAL4, both of which have previously been shown to drive expression in both nephrocyte populations (Lovric et al., 2017; Zhang et al., 2013a, 2013b; Zhuang et al., 2009). We used two different control RNAi transgenes targeting *GFP* or the testis-specific *Pkd2* (Köttgen et al., 2011). Compared to these control cells, depletion of *dHNF4* by two different RNAi lines (*dHNF4* knockdown [KD]) led to an increase in LD content, number, and size in all garland nephrocytes (Figures 1D–1G and S1A–S1E), which could also be observed using transmission electron microscopy (TEM; Figure 1H). The mitochondria of *dHNF4*-depleted cells showed reduced amounts of ATP5A, which is a subunit of the mitochondrial ATP synthase in the respiratory chain (Figures 1I, 1J, S1F, and S1G). Moreover, the accumulation of LDs in *dHNF4* KD cells could be suppressed by silencing *mdy* (Figures S2A and S2B), the ortholog of the endoplasmic reticulum (ER)-localized diacylglycerolacyltransferase 1 (DGAT1). In line with previous studies, these findings suggest that LD accumulation in nephrocytes is a result of reduced β-oxidation and subsequent DGAT1-dependent esterification of unmetabolized fatty acids into TAGs (Chitraju et al., 2017; Nguyen et al., 2017; Palanker et al., 2009).

Overexpression of dHNF4 Regulates LD in an Expression Level-Dependent Manner

In contrast to the KD, the overexpression of dHNF4 (dHNF4^{OE}) significantly decreased LD content in most nephrocytes (Figures 2A and 2B, inset 2), indicating an increased use of storage lipids for mitochondrial β-oxidation. Some cells, however, exhibited the opposite phenotype with strong LD accumulations, as shown in inset 1 and by the outliers in the graph of Figure 2B. LD^{+} cells showed an increase in the area covered by LDs that was, unlike *dHNF4* KD, not due to an increase in size but to an augmented LD number per cell volume (Figures 2C–2E). An enhanced ATP5A staining was observed in LD^{-} cells, but not in LD^{+}cells (Figures 2F and 2G), supporting the functional correlation between LD content and mitochondrial respiratory chain activity.

To test whether the different LD phenotypes were dependent on the expression level of dHNF4, we made use of the temperature dependency of the UAS-GAL4 system (Figure S3A) (Duffy, 2002). While low overexpression at 18°C showed complete LD depletion (Figures S3B–S3D), switching the temperature to 29°C at L3 larval stages led to strong lipid accumulation (Figures S3B–S3D) and low ATP5A levels in nephrocytes (Figure S3E). Hereafter, the three different expression levels are referred to as dHNF4^{lowOE} (18°C), dHNF4^{OE} (25°C), and dHNF4^{highOE} (29°C). While the temperature itself also increased LDs to some extent (Figure S3D), dHNF4^{highOE} cells always had more LDs than the controls and dHNF4^{lowOE} always had fewer LDs (Figures S3B–S3D). Moreover, co-expressing *mdy* RNAi with dHNF4 completely abolished LDs in both dHNF4^{OE} and dHNF4^{highOE} cells (Figures S2A and S2B), suggesting that similar to *dHNF4* KD, the increased LD amount, as in the KD, may be the result of unmetabolized fatty acids being diverted toward esterification and storage.

dHNF4^{OE} Leads to Nuclear Depletion of the Wild-Type Protomer

To better understand the phenotypic similarity between *dHNF4* RNAi and dHNF4^{OE}, we studied the localization of the overexpressed dHNF4 in nephrocytes, making use of its hemagglutinin (HA) tag. We identified a spectrum of localization patterns that matched the LD phenotypes seen in each overexpression condition: normal nuclear localization was only found in all dHNF4^{lowOE} and LD^{-} dHNF4^{OE} cells (Figures 3A and 3B), whereas enrichment at the lamin D^{+} nuclear periphery was detected for LD^{+} dHNF4^{OE} cells (Figure 3A) and dHNF4^{highOE} cells (Figures 3B and S3F). As HNF4A normally forms dimer, we also tested effects on the dHNF4-GFP reporter protein. We found that dHNF4^{OE} and dHNF4^{highOE} caused a mild and a strong reduction in nuclear dHNF4-GFP, respectively (Figures 3D and 3E). These data suggest that the phenotypic similarity between KD and dHNF4 overexpression may be explained by the loss of nuclear activity of the protomer expressed from the wild-type allele.

dHNF4^{OE} Causes Cytotoxic Effects

In dHNF4^{highOE} cells, the nuclear depletion of dHNF4 also correlated with the formation of cytoplasmic punctae that were positive for ubiquitin (Figures 3C and S3G). As ubiquitinated aggregates normally stimulate autophagy, we looked at autophagic flux by using the GFP-mCherry-Atg8 tandem reporter (Atg8 corresponds to LC3 in mammals) (Kimura et al., 2007). We observed an increase in Atg8-GFP-mCherry signal in dHNF4^{OE} (Figure S4A and insets). However, unlike the silencing of *ATP6AP2*, a subunit of the proton pump V-ATPase, this signal did not co-localize with GFP, suggesting that autophagic flux was enhanced (Figure S4A

(F) TEM sections of dHNF4-overexpressing nephrocytes. Arrows point to autophagic structures. Black arrowheads point to electron-dense aggregates. White arrowheads point to multi-layering of autophagosomal membranes. Scale bars, 1 μm (first and second panels) and 500 nm (third and fourth panels).
See also Figures S3 and S4.

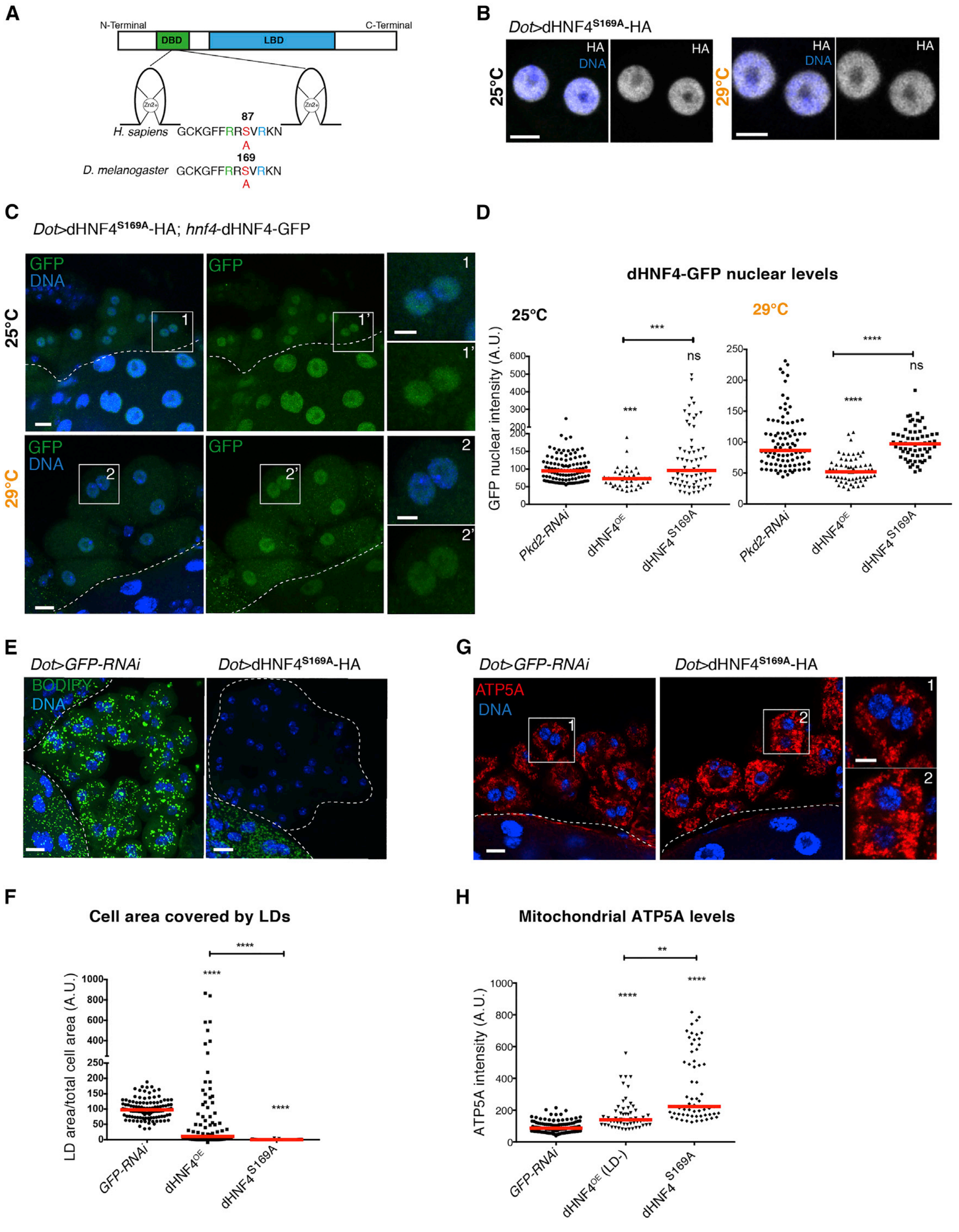

Figure 4. Blocking Degradation Signaling Prevents dHNF4-Induced Phenotypes

(A) Region of the DNA-binding domain between the two zinc fingers containing the S87 in humans, corresponding to S169 in flies.

(B) Confocal sections of representative nuclei stained for DNA and HA of nephrocytes overexpressing dHNF4^{S169A} at 25°C and 29°C. Scale bars, 5 μm.

(C) Z-projections of nephrocytes expressing dHNF4-GFP under the native HNF4 promoter and dHNF4^{S169A} at 25°C and 29°C, stained for GFP, HA, and DNA. Scale bars, 10 μm, insets 5 μm.

(D) Quantification of the average nuclear GFP intensity in (C), compared to that of control and dHNF4-overexpressing nephrocytes shown in Figure 3E. Values were normalized to control nephrocytes (three for 25°C and two for 29°C independent experiments, red lines: medians, one-way ANOVA test: ***$p < 0.001$, ****$p < 0.0001$, ns: non-significant).

(E) Z-projections showing control and dHNF4^{S169A}-overexpressing nephrocytes stained for LDs and DNA. Scale bars, 10 μm.

(legend continued on next page)

and insets). By performing TEM on dHNF4^{OE} cells, we found abundant cytoplasmic phagophores and autophagosomes, whose limiting membranes were often multilamellar in structure (Figure 3F). Numerous electron-dense structures could be detected inside and outside the autophagosomes, possibly representing ribosomes or the aggregates themselves (Figure 3F). Furthermore, we found in control cells a perinuclear compact ER decorated with ribosomes, while ER structures were difficult to detect in dHNF4^{OE} cells (Figure 3F). This was confirmed by immunostaining for the luminal ER marker protein di-sulfide isomerase (PDI) displaying an abnormal ER expansion for LD^{+} dHNF4^{OE} and dHNF4^{highOE} (Figures S4B and S4D), while the ER showed a normal perinuclear morphology in control cells and in dHNF4 KD and dHNF4^{lowOE} cells (Figures S4B and S4C).

The increase in autophagy and the ER abnormalities correlated well with the effects on the viability of nephrocytes and of the entire animal. With *sns*-GAL4, nephrocyte cytotoxicity became apparent already at L3 larval stage, causing a reduction in cell size and number in dHNF4^{OE} as compared to control (Figure S4E). With *Dot*-GAL4 but not *Sns*-GAL4, there was also a reduced animal viability, suggesting that this driver also expresses in cells besides nephrocytes that are essential for survival. While animals expressing dHNF4 at 29°C were dying already at L3 larval stages, there was an intermediate phenotype at 25°C with animals that were able to survive into adulthood, albeit with a loss of nephrocytes (Figure S4F). By contrast, the viability of nephrocytes and whole animals was unaffected in *dHNF4* KD- and dHNF4^{lowOE}-expressing animals (data not shown).

The data suggest the following scenario: the higher the expression of dHNF4, the more nuclear export and formation of cytosolic aggregates, which in turn lead to ER damage, autophagy, and eventually cell death. As none of these cytotoxic effects were found for *dHNF4* KD, we conclude that they are related to the cytosolic aggregates of dHNF4^{highOE}.

Blocking Degradation Signaling Prevents dHNF4^{highOE}-Induced Phenotypes

The nuclear export and turnover of HNF4A was previously shown to depend on PKC-dependent phosphorylation at the highly conserved serine 87 (S87) in the DBD (Figure 4A) (Sun et al., 2007). We therefore reasoned that suppressing the phosphorylation may also suppress the loss of nuclear HNF4 and cytotoxic effects. To address this, we attempted to suppress phosphorylation by introducing a serine-to-alanine conversion at the corresponding 169 position in the fly sequence (dHNF4^{S169A}; Figure 4A). To avoid any confounding positional effects, the UAS transgene was inserted at the same genomic position as the wild-type transgene. We first addressed the localization of this supposedly phospho-deficient form. dHNF4^{S169A} was nuclear at both 25°C and 29°C (Figure 4B), as was the dHNF4-GFP reporter (Figures 4C and 4D). Moreover, LD content was equally depleted as in dHNF4^{lowOE} cells (Figures 4E and 4F), and mitochondrial ATP5A was increased in dHNF4^{S169A} nephrocytes (Figures 4G and 4H), suggesting that dHNF4^{S169A} strongly promotes lipid catabolism. In addition, autophagy, ER morphology, and viability of nephrocytes were normal (Figures S6A–S6C). These data suggest that increasing nuclear dHNF4 levels by preventing nuclear export positively regulates lipid catabolism and mitochondrial function while suppressing any dominant-negative effects.

Renal Fanconi Syndrome-Associated *Drosophila* Mutation R167W Causes LD Accumulation and Mitochondrial Defects

Next, we investigated the impact of the MODY1 mutation R85W, which is uniquely associated with FRTS in humans. We compared its phenotypes to a nearby arginine-to-tryptophan substitution, R89W, that causes MODY1 without FRTS (Figure 5A) (Hamilton et al., 2014). Previously, the crystal structure of human HNF4A had shown that both R85 and R89 are in close proximity to DNA and that replacing either of the arginines with tryptophan could impair DNA binding (Bogan et al., 2000). We confirmed this hypothesis by performing an electrophoretic mobility shift assay (EMSA) in COS-7 cells, showing that while HNF4 wild-type (WT) and S87A readily bound to a DNA probe with HNF4A target motif (Jiang et al., 1995), the binding of HNF4 R85W and R89W was strongly reduced (Figure 5B).

In flies, the arginines in positions 85 and 89 correspond to the arginines in positions 167 and 171 (Figure 5A). We generated two UAS transgenes harboring the two mutations, respectively (hereafter called dHNF4$^{R167W/FRTS}$ and dHNF4$^{R171W/MODY}$). Nephrocytes expressing dHNF4$^{R167W/FRTS}$ displayed either an enrichment of the HA staining at the nuclear periphery or a very weak staining in the nucleus (Figure 5C). Nuclear localization of dHNF4-GFP was completely lost, accompanied by a very strong increase in GFP^{+} puncta in the cytosol (Figures 5D and 5E). Accordingly, these cells displayed a strong increase in LDs (Figures 6A and 6B), which could be fully suppressed by *mdy* KD (Figures S6A and S6B). In addition, mitochondrial ATP5A staining was strongly decreased (Figures 6C and 6D), while autophagy was increased and ER morphology strongly disturbed (Figures 6E and S6G). When using the *Sns*-GAL4 driver nephrocyte, loss was already apparent at early larval stages (Figure 6F). When using the *dot*-GAL4 driver, there was also a strongly reduced animal lethality. In contrast to wild-type dHNF4, these phenotypes could even be observed at 18°C (Figures S6D and S6F), indicating that the dominant-negative effects of dHNF4$^{R167W/FRTS}$ are more severe than they are for dHNF4^{OE}.

The expression of dHNF4$^{R171W/MODY}$ caused no dominant-negative effects. Instead, the situation was highly similar to the expression of the phosphorylation-deficient dHNF4^{S169A}: there

(F) Quantification of the cell area occupied by lipid droplets per cell in the genotypes shown in (E) compared to the cell area of nephrocytes expressing dHNF4 shown in Figure 2B. Values were normalized to control nephrocytes (three independent experiments, red lines: medians, one-way ANOVA test, ****$p < 0.0001$.
(G) Confocal sections of control and dHNF4^{S169A}-overexpressing nephrocytes, stained for ATP5A and DNA. Scale bars, 10 μm, insets 5 μm.
(H) Quantification of the ATP5A staining intensity shown in (G) compared to the staining intensity of nephrocytes expressing dHNF4 (LD^{-}) shown in Figure 2G. Values were normalized to the average value in control nephrocytes (three for dHNF4^{S169A} and five for dHNF4^{OE} independent experiments, red lines: medians, one-way ANOVA test: ****$p < 0.0001$, **$p < 0.01$).
See also Figure S5.

OPEN ACCESS
CellPress

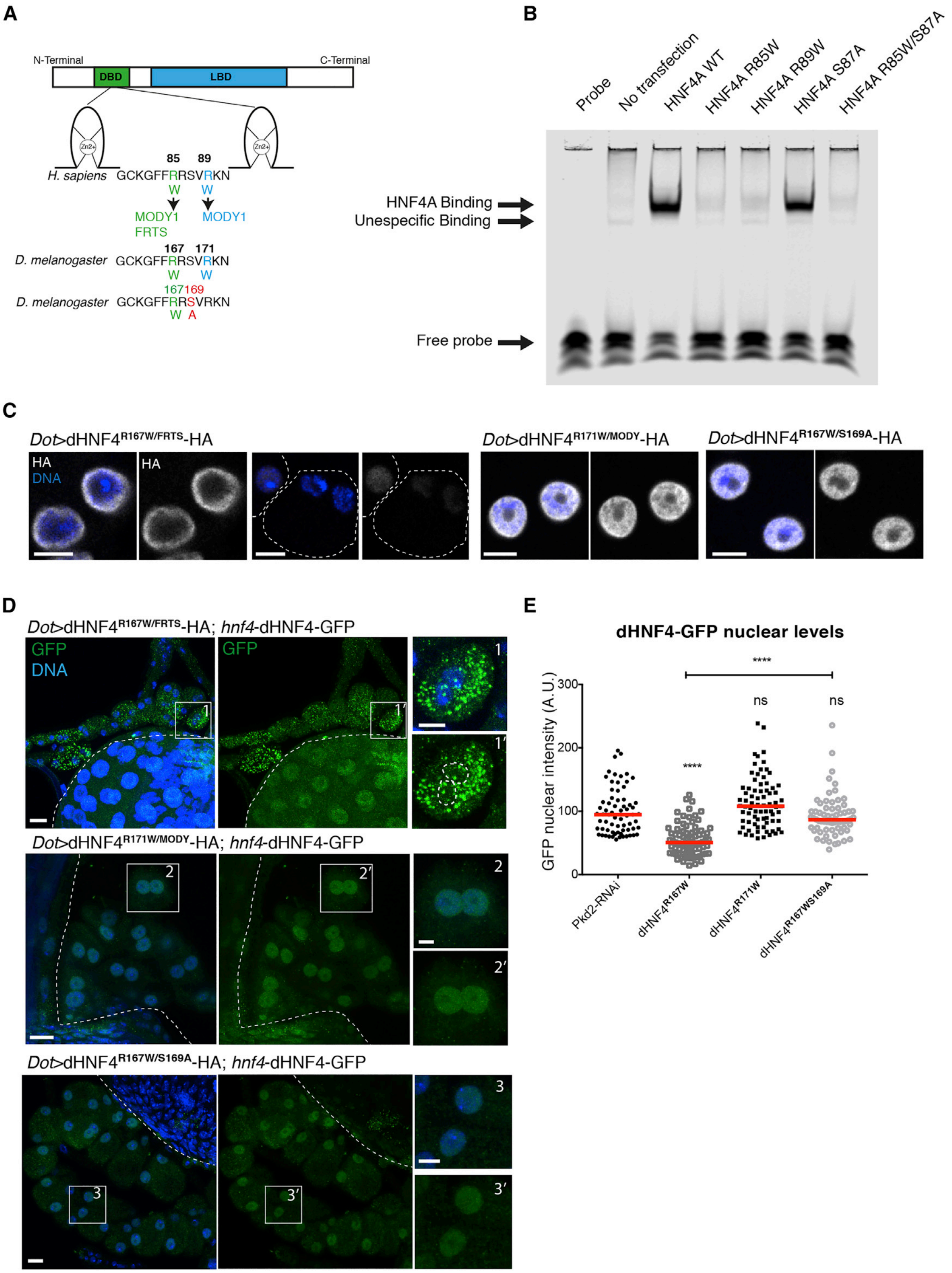

Figure 5. Renal Fanconi Syndrome-Associated Mutation dHNF4 (p.R167W) Causes Nuclear Depletion of the Wild-Type Protomer That Can Be Rescued by Blocking Degradation Signaling

(A) Region of the DNA-binding domain between the two zinc fingers containing the human R85W mutation causing both MODY1 and Fanconi renotubular syndrome (FRTS) and the R89W mutation causing MODY1, corresponding to R167W and R171W, respectively, in flies. Below is the dHNF4 double mutant containing R167W/FRTS and S169A mutations (dHNF4$^{R167W/S169A}$-HA).

(B) EMSA of COS-7 nuclear extracts containing overexpressed HNF4A WT and mutants with the ApoAI site A probe.

(legend continued on next page)

was a normal nuclear distribution of both the transgene and dHNF4-GFP (Figures 5C–5E). Mitochondria staining, ER morphology, and viability were normal (Figures 6C–6F). Similar observations were made at 29°C (Figures S6C and S6E). The only difference from dHNF4^{S169A} was that for dHNF4$^{R171W/MODY}$, the LD content was only reduced and not fully eliminated (Figure 6A), which is in line with the reduced DNA binding in the EMSA assay (Figure 5B).

S169A Partially Rescues R167W/FRTS Phenotypes

To prevent phosphorylation in both mutants, we created double mutants of S87A and R167/FRTS and R171W/MODY (Figure 5A). As expected, the nuclear localization of R171W/MODY was unaffected by the additional mutation (not shown). However, the expression of R167W/S169A double mutant showed a strong increase in nuclear levels compared to R167W alone (Figure 5C). The nuclear localization of dHNF4-GFP reporter (Figures 5D and 5E), the ER morphology (Figure 6E), and the autophagy levels (Figure S6G) were entirely restored, suggesting suppressed dominant-negative effects. Moreover, S169A caused a striking restoration of the viability of the whole animal when using *Dot*-GAL4 (data not shown) and of nephrocytes when using *Sns*-GAL4 as drivers (Figure 6F). Although mitochondrial ATP5A levels seemed normalized (Figures 6C and 6D), LD content remained high in the R167W/S169A double mutant (Figures 6A and 6B), suggesting that the dephosphorylation cannot rescue the decreased transcriptional activity. This was supported by the finding that the R85W/S87A double mutant showed almost no detectable DNA binding in the EMSA shift assay (Figure 5B). Therefore, our data demonstrate that preventing the mutant protein from being phosphorylated at S169 and expelled from the nucleus for degradation strongly diminishes all dominant-negative effects of dHNF4 without rescuing lipid metabolism defects (Table S1).

R85W/FRTS Mutation Causes Mitochondrial Dysfunction in Mouse Reprogrammed Renal Epithelial-like Cells

Finally, to validate the effects of the FRTS mutation in a mammalian model, we chose a direct reprogramming approach, called induced renal epithelial cells (iRECs) (Kaminski et al., 2016), in which HNF4A is one of three transcription factors inducing renal epithelial cell fate (Figure 7A). In this system, HNF4A is particularly important for the expression of several proximal tubular markers (Kaminski et al., 2016), which is in agreement with *in vivo* mouse studies (Marable et al., 2018). Embryonic fibroblasts were isolated from mouse embryonic fibroblasts (MEFs) harboring Cre-recombinase under the control of the renal tubule-specific cadherin-16 promoter (Cdh16/Ksp-Cre) and the tandem dimer Tomato red/EGFP (tdTomato/EGFP) dual fluorescent reporter (Shao et al., 2002). Upon lentiviral transduction with Pax8, Hnf1b, and HNF4A (wild-type or R85W/FRTS), cells that had switched from Tomato to EGFP expression and thus had acquired renal cell fate were isolated by fluorescence-activated cell sorting (FACS) and then functionally characterized (Figure 7A).

Reprogramming to iRECs was achieved with both the wild-type HNF4A and the R85W/FRTS mutation at similar rates (0.66% and 0.69%, respectively). We detected lower mRNA levels of HNF4A R85W expression compared to wild-type (Figure 7B). Using immunostaining, we also found lower nuclear staining for HNF4A R85W/FRTS protein (Figures 7C and 7D). However, the overall changes to the transcriptional profile analyzed by RNA sequencing (RNA-seq) were remarkably similar between iRECs reprogrammed using wild-type HNF4A and HNF4A R85W/FRTS, suggesting that the R85W/FRTS mutation retains reprogramming activity and elicits overall similar changes to the gene expression profile from fibroblasts to iRECs (Figure 7E). Cluster mapping and Gene Ontology (GO)-term enrichment analysis revealed that genes related to mitochondrial function were induced by reprogramming using wild-type HNF4A, but they had a significantly reduced expression in cells reprogrammed using HNF4A R85W/FRTS (Figures 7F, 7G, S7A, and S7B).

For functional validation, we analyzed the cells with Seahorse XF96 mitochondrial flux analyzer. iRECs reprogrammed with R85W/FRTS showed decreased ATP production and basal respiration together with a strongly impaired maximal respiration (Figure 7H). Although R85W/FRTS iRECs were able to use fatty acids for basal respiration, the treatment of the cells with the mitochondrial fatty acid uptake inhibitor etomoxir showed that the oxygen consumption rate via the oxidation of fatty acids was relatively lower compared to iREC WT cells (Figure 7I). Accordingly, due to the change in mitochondrial function, LDs were increased in iREC R85W compared to iRECs WT (Figures 7J and 7K), confirming the nephrocyte findings in these proximal tubule-like cells.

DISCUSSION

HNF4A is specifically expressed in the kidney in proximal tubules and is required for the terminal differentiation of proximal tubular cells (Jiang et al., 2003; Marable et al., 2018). However, out of all of the known mutations, only the R85W mutation causes FRTS (Hamilton et al., 2014). Using fly nephrocytes as a model for proximal tubules, we show here that both dHNF4 KD and overexpression of the FRTS mutation (R167W in flies) causes mitochondrial alterations and LD accumulation. By using a dHNF4-GFP as a reporter protein, we show that the FRTS mutation provokes the nuclear export of the wild-type protomer in a

(C) Confocal sections of representative nuclei stained for DNA and HA of nephrocytes overexpressing dHNF4$^{R167W/FRTS}$-HA, dHNF4$^{R171W/MODY}$-HA, and dHNF4$^{R167W/S169A}$-HA. Scale bars, 5 μm.

(D) Z-projections of nephrocytes expressing dHNF4-GFP under the native *dHNF4* promoter together with dHNF4$^{R167W/FRTS}$-HA, dHNF4$^{R171W/MODY}$-HA, and dHNF4$^{R167W/S169A}$-HA, stained for GFP and DNA. Scale bars, 10 μm, insets 5 μm.

(E) Quantification of the average nuclear GFP intensity in (A). Values were normalized to control nephrocytes (four independent experiments for dHNF4$^{R167W/FRTS}$-HA and dHNF4$^{R171W/MODY}$-, three for dHNF4$^{R167W/S169A}$-HA, red bars: medians, one-way ANOVA test: ****p < 0,0001, ns: non-significant).

See also Figure S6.

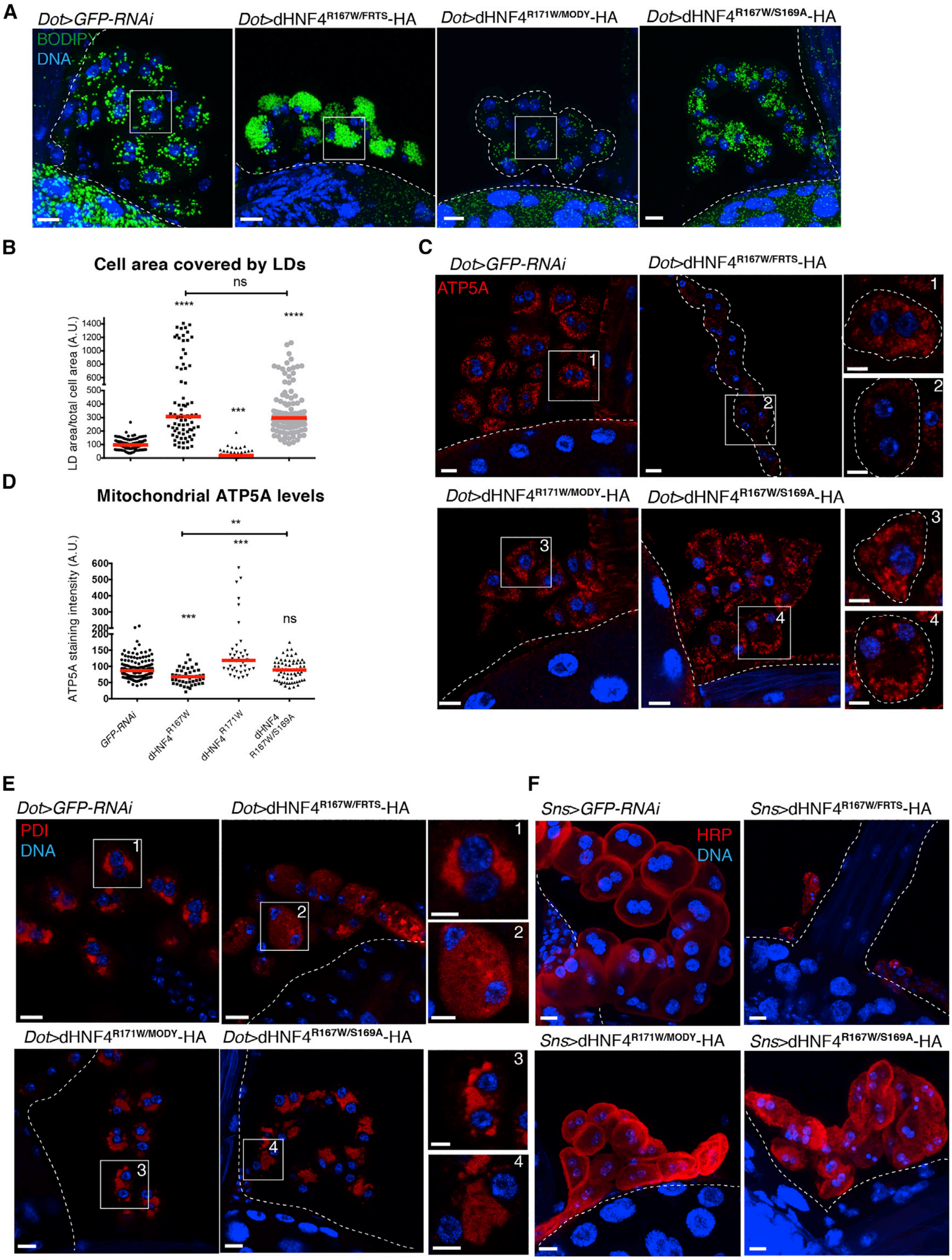

Figure 6. dHNF4$^{R167W/FRTS}$ Causes LD Accumulation, Mitochondrial Defects, and Cytotoxic Effects That Can Be Rescued by Blocking Degradation Signaling

(A) Z-projections showing LD and DNA staining in control, dHNF4$^{R167W/FRTS}$-, dHNF4$^{R171W/MODY}$-, or dHNF4$^{R167W/S169A}$-overexpressing nephrocytes. Scale bars, 10 μm.

(B) Quantification of the cell area occupied by LDs per cell in the genotypes shown in (A) (five independent experiments for dHNF4$^{R167W/FRTS}$, three for dHNF4$^{R171W/MODY}$ and dHNF4$^{R167W/S169A}$, red lines: medians, one-way ANOVA test: ***$p < 0.001$, ****$p < 0.0001$, ns: non-significant).

(legend continued on next page)

dominant-negative manner, possibly explaining the similarity to the KD. The nuclear export is additionally associated with cytoplasmic aggregate formation, increased autophagy, severe ER morphology changes, and eventually cell death. All of these phenotypes are not seen in the KD, suggesting that they are the result of the aggregate formation. However, they could be observed when expressing wild-type dHNF4 at high levels, suggesting dominant-negative effects in this condition as well. For both dHNF4$^{R167W/FRTS}$ and dHNF4^{highOE}, these effects could be rescued by the increase in nuclear levels through dephosphorylation at S169.

Crucial for the understanding of the investigated mutations are structural studies of the HNF4A homodimer/DNA complex, in which the homodimer forms multiple domain-domain junctions and a convergence zone or "nerve center," in which the two LBDs, the upstream positioned DBD, and the hinge region meet. S87 maps precisely to this center, leading to unfavorable charge repulsion upon phosphorylation and disengagement of the quaternary structure needed for DNA binding (Chandra et al., 2013). In another nuclear receptor, constitutive androstane receptor (CAR), the corresponding phosphorylation leads to the formation of inactive homodimers (Shizu et al., 2017), providing support for this model. As R85 is in close contact with the DNA bases and backbone of the dipartite DR1 element in both upstream and downstream protomers of the homodimer (Chandra et al., 2013), the lack of DNA binding in the R85W mutant may promote phosphorylation-dependent conversion of active to inactive homodimers and, finally, nuclear export of both mutant and wild-type protomers.

By contrast, the effects of the MODY mutation R171W resembled those of the phosphorylation-deficient S169A mutant, which is supported by two phosphorylation prediction algorithms (https://scansite4.mit.edu/4.0/#home and http://www.cbs.dtu.dk/services/NetPhos/), showing that R171W/MODY (or R89W/MODY) but not R167W/FRTS (or R85W/FRTS) reduces the probability for phosphorylation at S169 (or S87). In addition, the available HNF4A structure shows that R89 is closer to the S87 convergence zone compared to R85 (Chandra et al., 2013), which may explain that at the R171 (or R89) position, the substitution with the bulky tryptophan could interfere with the S169 (or the mammalian S87) phosphorylation site. Unlike for R167W (or R85W), the reduced DNA binding of R171W (or R89W) may therefore not result in phosphorylation-dependent dominant-negative effects, which is consistent with the haploinsufficiency that has been proposed for most MODY1 mutations (Ferrer, 2002). The reverse conclusion would be that haploinsufficiency alone is not enough to cause FRTS and that dominant-negative effects are required in addition.

As a consequence of the nuclear export due to the dominant-negative mechanism, we uncovered cytotoxic effects that were not seen in the dHNF4 KD and hence are most likely linked with the aggregation of potentially misfolded proteins in the cytoplasm. Our co-labeling with the nuclear envelope marker lamin D shows that the aggregate formation already commences during nuclear export. It can therefore be assumed that the small nuclear pore size causes an accumulation of dHNF4 beneath the nuclear envelope, which in turn favors aggregate formation. Although the order of cytosolic events is not entirely clear, we speculate that the aggregates first pose a challenge for the clearance capacity of the proteasome, which is the normal site of HNF4A degradation (Sun et al., 2007). As a result, the ER may help by translocating chaperones into the cytoplasm to stimulate autophagy, as has been described for BiP and PDI (Cha-Molstad et al., 2015). Consequently, the ability of the ER to address its own misfolded membrane-bound or luminal proteins could be weakened, thereby causing ER stress. Previously, cytoplasmic aggregates due to dominant-negative mutations or polyQ expansions have been observed in two other nuclear receptors, the thyroid receptor and the androgen receptor, respectively (Bunn et al., 2001). In the latter case, the cytoplasmic aggregates were also associated with ER stress (Thomas et al., 2005).

While dHNF4 has been shown to act as a regulator of mitochondria gene expression in the fly (Barry and Thummel, 2016; Bülow et al., 2018; Palanker et al., 2009), the role of mammalian HNF4A in regulating mitochondrial function has so far been poorly investigated. Using the iREC system, we show here that the R85W mutation affects mitochondria by decreasing the transcription of genes for mitochondrial structure and function. This was accompanied by a reduction in β-oxidation, leading to LD accumulation. As the HNF4A R85W also showed lower mRNA levels, we were unable to assess the contribution of any dominant-negative effects in this model. Also, we could not directly measure any effects of HNF4A R85W on DNA binding as we did in the COS-7 cell system. Nevertheless, both the nephrocyte and iREC results suggest that the effect on mitochondria could be one of the reasons why HNF4A R85W affects the fatty acid-consuming proximal tubules (Kang et al., 2015). In this manner, HNF4A-associated FRTS would be related to mitochondriopathies with isolated Fanconi syndrome or Fanconi syndrome as renal manifestation in more widespread disease (Klootwijk et al., 2014; O'Toole, 2014; Reichold et al., 2018). Given that HNF4A may be regulated by free fatty acids, it would be interesting to explore how extensively HNF4A is controlled by the reabsorption of exogenous fatty acids in proximal tubules (or nephrocytes) and whether fatty acid re-esterification and storage in LDs counteracts HNF4A activation.

(C) Confocal sections showing nephrocytes expressing *GFP-RNAi*, dHNF4$^{R167W/FRTS}$, HNF4$^{R171W/MODY}$, or dHNF4$^{R167W/S169A}$, stained for ATP5A and DNA. Scale bars, 10 μm, insets 5 μm.

(D) Quantification of the ATP5A staining intensity shown in (C). Values were normalized to the average value in control nephrocytes (three independent experiments, red lines: medians, one-way ANOVA test $^{**}p < 0.01$, $^{***}p < 0.001$, $^{****}p < 0.0001$, ns: non-significant).

(E) Confocal sections showing PDI and DNA staining in nephrocytes expressing *GFP-RNAi*, dHNF4$^{R167W/FRTS}$, dHNF4$^{R171W/MODY}$, or dHNF4$^{R167W/S169A}$. Scale bars, 10 μm, insets: 5 μm.

(F) Z-projections of larval garland nephrocytes expressing *GFP-RNAi*, dHNF4$^{R167W/FRTS}$, dHNF4$^{R171W/MODY}$, or dHNF4$^{R167W/S169A}$ driven by *Sns*-GAL4 and stained for DNA and for HRP-Cy3 staining nephrocyte membranes. Scale bars, 10 μm.

See also Figure S6 and Table S1.

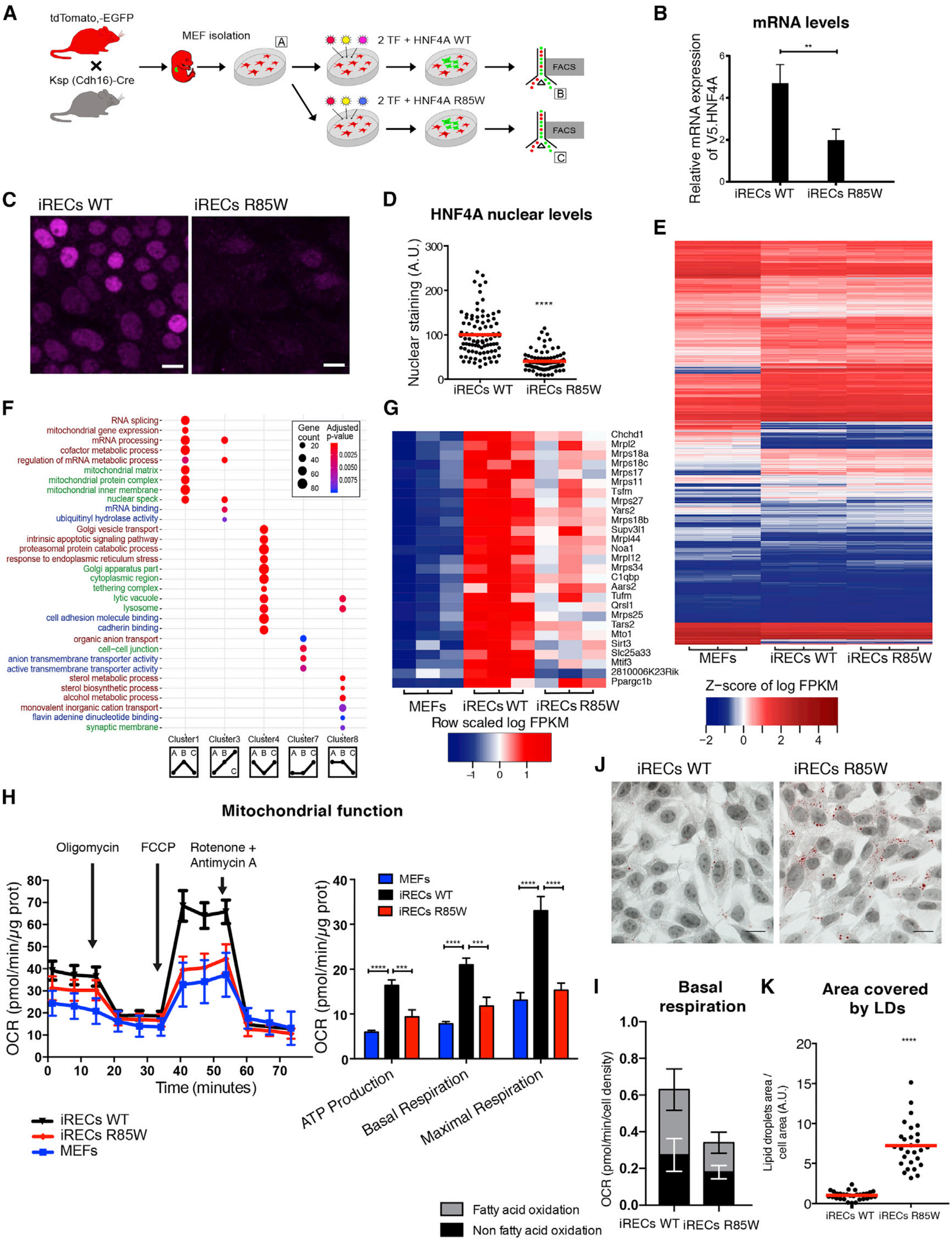

Figure 7. Direct Reprogramming of Mouse Embryonic Fibroblasts to iRECs Using HNF4A R85W

(A) Schematic representation of the reprogramming strategy. Mouse embryonic fibroblasts (MEFs) were isolated from the limbs of Ksp-Cre/dtTomato;EGFP E13.5 pups and transduced with the indicated transcription factor cocktails.

(B) Relative mRNA expression level of exogenous HNF4A in iRECs HNF4A^{WT}-V5 and iRECs HNF4A^{R85W}-V5. qPCR primers encompass the V5 region and parts of the coding sequence of transduced HNF4A. Values were normalized to the mTBP housekeeping gene (Mann-Whitney t test: **p < 0.01).

(C) Immunostainings of the V5-tag in HNF4A^{WT}-V5 and HNF4A^{R85W}-V5 iRECs. Scale bar, 10 μm.

(D) Quantification of the nuclear staining normalized to the average value of iRECs WT (three independent experiments, red lines: medians, Mann-Whitney t test, ****p < 0.0001).

(legend continued on next page)

In conclusion, our findings establish HNF4A as a master regulator of lipid metabolism in nephrocytes with high relevance for proximal tubular cells. We further provide insight into the molecular basis of HNF4A-associated FRTS that has remained enigmatic since the identification of the first patient with the R85W mutation (Hamilton et al., 2014). Our results suggest a rationale for treatments aimed at preventing the nuclear export of HNF4A in such patients. As serine phosphorylation in the DBD has been shown to be conserved in many nuclear hormone receptors (Sun et al., 2007), blocking this phosphorylation as a means to increase nuclear activity should also have more general implications.

STAR★METHODS

Detailed methods are provided in the online version of this paper and include the following:

- KEY RESOURCES TABLE
- LEAD CONTACT AND MATERIALS AVAILABILITY
- EXPERIMENTAL MODEL AND SUBJECT DETAILS
  - Fly strains
  - Cell lines
- METHOD DETAILS
  - Generation of fly strains
  - Immunohistochemistry, lipid droplet labeling and tissue imaging
  - X-gal staining
  - Transmission electron microscopy
  - Cloning and lentivirus production
  - EMSA
  - iREC generation and immunocytochemistry
  - Mitochondrial activity measurements
  - RNA sequencing and qPCR
- QUANTIFICATION AND STATISTICAL ANALYSIS
  - Image analysis
  - Statistics
  - RNA-seq data analysis
- DATA AND CODE AVAILABILITY

SUPPLEMENTAL INFORMATION

Supplemental Information can be found online at https://doi.org/10.1016/j.celrep.2019.11.066.

ACKNOWLEDGMENTS

We are grateful to Tor Erik Rusten, Carl Thummel, and the Developmental Studies Hybridoma Bank (DSHB) for providing antibodies. We thank Gabor Juhasz, Susan Abmayr, Zhe Han, the Bloomington Drosophila Stock Center, FlyORF, and the Vienna *Drosophila* RNAi Center (VDRC) for providing flies. We thank Christine Schmitt and Jacomina Krijnse Locker from the Ultrastructural BioImaging facility of the Pasteur Institute for performing TEM; the Cell Imaging platform, Structure Fédérative de Recherche de Necker, Inserm US 24 for assistance with microscopy; and Ivan Nemazanyy from the Metabolism platform, Structure Fédérative de Recherche de Necker, for performing the Seahorse experiments. We are grateful to Robin Klemm, Robert Ernst, Tobias Hermle, and Philippe Chavrier for critically reading the manuscript. The work has been supported by the ATIP-Avenir program of the Fondation Bettencourt-Schueller (Liliane Bettencourt Chair of Developmental Biology) as well as state funding by the Agence Nationale de la Recherche (ANR) under the "Investissements d'Avenir" program (ANR-10-IAHU-01) and the NEPHROFLY (ANR-14-ACHN-0013) grant to M.S. A.P.-M. is funded by a postdoctoral fellowship from the Fondation pour la Recherche Médicale (FRM-SPF20170938629). This project has received funding from the European Research Council (ERC) under the European Union's Horizon 2020 research and innovation program grant agreement 804474 to S.L. It is also supported by the Emmy Noether Programme (LI1817/2-1 to S.L.) and Project B07 of the collaborative research initiative (SFB 1140 to S.L.) by the German Research Foundation (DFG). E.D.K. and R.K. were supported by the St. Peter's Trust for Kidney, Bladder, and Prostate Research.

AUTHOR CONTRIBUTIONS

V.M. designed the project and the experiments, performed the experiments, analyzed the results, and wrote the manuscript. A.P.-M. performed the experiments and analyzed the results on iRECs. G.L.M. performed the experiments on flies. R.P. generated iRECs and performed qPCR. R.P., K.G., and S.L. generated and analyzed the RNA-seq data. E.D.K., A.K., R.K. and S.L. provided material and critical discussions. M.S. conceived and supervised the study, contributed to the experimental design, and wrote the manuscript.

DECLARATION OF INTERESTS

The authors declare no competing interests.

Received: April 29, 2019
Revised: October 8, 2019
Accepted: November 15, 2019
Published: December 24, 2019

REFERENCES

Afgan, E., Baker, D., van den Beek, M., Blankenberg, D., Bouvier, D., Čech, M., Chilton, J., Clements, D., Coraor, N., Eberhard, C., et al. (2016). The Galaxy

(E) Heatmap of the significant differentially expressed genes (DEGs) (N = 10,963) between all three analyzed conditions. Each row represents a gene. Each column represents one of three biological replicates per reprogramming condition. FPKM, fragments per kilobase million.
(F) GO overrepresentation analysis using clusterProfiler. In the schematic depiction of the cluster categories, the first dot represents MEFs (A), the second dot represents iRECs HNF4a WT (B), and the third dot represents iRECs HNF4a R85W (C; see also A). Not more than five of the most significant terms from each biological domain were plotted for clusters 1, 3, 4, 7, and 8. The labels for biological processes are marked in red, molecular function in blue, and cellular component in green. The size of the spheres corresponds to the number of genes included and the color to the adjusted p value.
(G) Heatmap of genes representing mitochondrial gene expression.
(H) Left: Seahorse XF Cell Mito Stress Test profile. OCR, oxygen consumption rate. Arrows indicate time for drug injection. A representative experiment is shown. Values represent medians ± SEMs of triplicates. Right: Seahorse parameters calculated as indicated in Method Details. Values are shown as medians ± SEMs of replicates from three independent experiments. One-way ANOVA with post hoc Tukey's test, ***$p < 0.001$, ****$p < 0.0001$.
(I) Basal respiration attributed to fatty acid oxidation using etomoxir calculated as indicated in Method Details. Values are shown as medians ± SEMs of replicates from three independent experiments.
(J) Bright-field images of lipid droplets stained with oil red O and counterstained with hematoxylin. Scale bar, 10 μm.
(K) Quantification of the cell area occupied by lipid droplets (three independent experiments, red lines: medians, Mann-Whitney t test: ****$p < 0.0001$).
See also Figure S7.

platform for accessible, reproducible and collaborative biomedical analyses: 2016 update. Nucleic Acids Res. *44* (*W1*), W3–W10.

Anık, A., Çatlı, G., Abacı, A., and Böber, E. (2015). Maturity-onset diabetes of the young (MODY): an update. J. Pediatr. Endocrinol. Metab. *28*, 251–263.

Barry, W.E., and Thummel, C.S. (2016). The Drosophila HNF4 nuclear receptor promotes glucose-stimulated insulin secretion and mitochondrial function in adults. eLife *5*, e11183.

Bobulescu, I.A. (2010). Renal lipid metabolism and lipotoxicity. Curr. Opin. Nephrol. Hypertens. *19*, 393–402.

Bogan, A.A., Dallas-Yang, Q., Ruse, M.D., Jr., Maeda, Y., Jiang, G., Nepomuceno, L., Scanlan, T.S., Cohen, F.E., and Sladek, F.M. (2000). Analysis of protein dimerization and ligand binding of orphan receptor HNF4alpha. J. Mol. Biol. *302*, 831–851.

Brand, A.H., and Perrimon, N. (1993). Targeted gene expression as a means of altering cell fates and generating dominant phenotypes. Development *118*, 401–415.

Bülow, M.H., Wingen, C., Senyilmaz, D., Gosejacob, D., Sociale, M., Bauer, R., Schulze, H., Sandhoff, K., Teleman, A.A., Hoch, M., and Sellin, J. (2018). Unbalanced lipolysis results in lipotoxicity and mitochondrial damage in peroxisome-deficient *Pex19* mutants. Mol. Biol. Cell *29*, 396–407.

Bunn, C.F., Neidig, J.A., Freidinger, K.E., Stankiewicz, T.A., Weaver, B.S., McGrew, J., and Allison, L.A. (2001). Nucleocytoplasmic shuttling of the thyroid hormone receptor alpha. Mol. Endocrinol. *15*, 512–533.

Cattin, A.L., Le Beyec, J., Barreau, F., Saint-Just, S., Houllier, A., Gonzalez, F.J., Robine, S., Pinçon-Raymond, M., Cardot, P., Lacasa, M., and Ribeiro, A. (2009). Hepatocyte nuclear factor 4alpha, a key factor for homeostasis, cell architecture, and barrier function of the adult intestinal epithelium. Mol. Cell. Biol. *29*, 6294–6308.

Cha-Molstad, H., Sung, K.S., Hwang, J., Kim, K.A., Yu, J.E., Yoo, Y.D., Jang, J.M., Han, D.H., Molstad, M., Kim, J.G., et al. (2015). Amino-terminal arginylation targets endoplasmic reticulum chaperone BiP for autophagy through p62 binding. Nat. Cell Biol. *17*, 917–929.

Chandra, V., Huang, P., Potluri, N., Wu, D., Kim, Y., and Rastinejad, F. (2013). Multidomain integration in the structure of the HNF-4α nuclear receptor complex. Nature *495*, 394–398.

Chitraju, C., Mejhert, N., Haas, J.T., Diaz-Ramirez, L.G., Grueter, C.A., Imbriglio, J.E., Pinto, S., Koliwad, S.K., Walther, T.C., and Farese, R.V., Jr. (2017). Triglyceride Synthesis by DGAT1 Protects Adipocytes from Lipid-Induced ER Stress during Lipolysis. Cell Metab. *26*, 407–418.e3.

Clemente, M., Vargas, A., Ariceta, G., Martínez, R., Campos, A., and Yeste, D. (2017). Hyperinsulinaemic hypoglycaemia, renal Fanconi syndrome and liver disease due to a mutation in the *HNF4A* gene. Endocrinol. Diabetes Metab. Case Rep. *2017*, 16-0133.

Duda, K., Chi, Y.I., and Shoelson, S.E. (2004). Structural basis for HNF-4alpha activation by ligand and coactivator binding. J. Biol. Chem. *279*, 23311–23316.

Duffy, J.B. (2002). GAL4 system in Drosophila: a fly geneticist's Swiss army knife. Genesis *34*, 1–15.

Duncan, S.A., Navas, M.A., Dufort, D., Rossant, J., and Stoffel, M. (1998). Regulation of a transcription factor network required for differentiation and metabolism. Science *281*, 692–695.

Fang, B., Mane-Padros, D., Bolotin, E., Jiang, T., and Sladek, F.M. (2012). Identification of a binding motif specific to HNF4 by comparative analysis of multiple nuclear receptors. Nucleic Acids Res. *40*, 5343–5356.

Ferrer, J. (2002). A genetic switch in pancreatic beta-cells: implications for differentiation and haploinsufficiency. Diabetes *51*, 2355–2362.

Hamilton, A.J., Bingham, C., McDonald, T.J., Cook, P.R., Caswell, R.C., Weedon, M.N., Oram, R.A., Shields, B.M., Shepherd, M., Inward, C.D., et al. (2014). The HNF4A R76W mutation causes atypical dominant Fanconi syndrome in addition to a β cell phenotype. J. Med. Genet. *51*, 165–169.

Helmstädter, M., and Simons, M. (2017). Using Drosophila nephrocytes in genetic kidney disease. Cell Tissue Res. *369*, 119–126.

Improda, N., Shah, P., Guemes, M., Gilbert, C., Morgan, K., Sebire, N., Bockenhauer, D., and Hussain, K. (2016). Hepatocyte Nuclear Factor-4 Alfa Mutation Associated with Hyperinsulinaemic Hypoglycaemia and Atypical Renal Fanconi Syndrome: Expanding the Clinical Phenotype. Horm. Res. Paediatr. *86*, 337–341.

Jiang, G., Nepomuceno, L., Hopkins, K., and Sladek, F.M. (1995). Exclusive homodimerization of the orphan receptor hepatocyte nuclear factor 4 defines a new subclass of nuclear receptors. Mol. Cell. Biol. *15*, 5131–5143.

Jiang, S., Tanaka, T., Iwanari, H., Hotta, H., Yamashita, H., Kumakura, J., Watanabe, Y., Uchiyama, Y., Aburatani, H., Hamakubo, T., et al. (2003). Expression and localization of P1 promoter-driven hepatocyte nuclear factor-4α (HNF4α) isoforms in human and rats. Nucl. Recept. *1*, 5.

Kaminski, M.M., Tosic, J., Kresbach, C., Engel, H., Klockenbusch, J., Müller, A.L., Pichler, R., Grahammer, F., Kretz, O., Huber, T.B., et al. (2016). Direct reprogramming of fibroblasts into renal tubular epithelial cells by defined transcription factors. Nat. Cell Biol. *18*, 1269–1280.

Kang, H.M., Ahn, S.H., Choi, P., Ko, Y.A., Han, S.H., Chinga, F., Park, A.S., Tao, J., Sharma, K., Pullman, J., et al. (2015). Defective fatty acid oxidation in renal tubular epithelial cells has a key role in kidney fibrosis development. Nat. Med. *21*, 37–46.

Kimura, S., Noda, T., and Yoshimori, T. (2007). Dissection of the autophagosome maturation process by a novel reporter protein, tandem fluorescent-tagged LC3. Autophagy *3*, 452–460.

Klootwijk, E.D., Reichold, M., Helip-Wooley, A., Tolaymat, A., Broeker, C., Robinette, S.L., Reinders, J., Peindl, D., Renner, K., Eberhart, K., et al. (2014). Mistargeting of peroxisomal EHHADH and inherited renal Fanconi's syndrome. N. Engl. J. Med. *370*, 129–138.

Klootwijk, E.D., Reichold, M., Unwin, R.J., Kleta, R., Warth, R., and Bockenhauer, D. (2015). Renal Fanconi syndrome: taking a proximal look at the nephron. Nephrol. Dial. Transplant. *30*, 1456–1460.

Ko, H.L., Zhuo, Z., and Ren, E.C. (2019). HNF4alpha Combinatorial Isoform Heterodimers Activate Distinct Gene Targets that Differ from Their Corresponding Homodimers. Cell Rep. *26*, 2549–2557.e3.

Köttgen, M., Hofherr, A., Li, W., Chu, K., Cook, S., Montell, C., and Watnick, T. (2011). Drosophila sperm swim backwards in the female reproductive tract and are activated via TRPP2 ion channels. PLoS One *6*, e20031.

Kumar, L., and Futschik, M.E. (2007). Mfuzz: a software package for soft clustering of microarray data. Bioinformation *2*, 5–7.

Liu, J., Shen, Q., Li, G., and Xu, H. (2018). HNF4A-related Fanconi syndrome in a Chinese patient: a case report and review of the literature. J. Med. Case Reports *12*, 203.

Lovric, S., Goncalves, S., Gee, H.Y., Oskouian, B., Srinivas, H., Choi, W.I., Shril, S., Ashraf, S., Tan, W., Rao, J., et al. (2017). Mutations in sphingosine-1-phosphate lyase cause nephrosis with ichthyosis and adrenal insufficiency. J. Clin. Invest. *127*, 912–928.

Maestro, M.A., Cardalda, C., Boj, S.F., Luco, R.F., Servitja, J.M., and Ferrer, J. (2007). Distinct roles of HNF1beta, HNF1alpha, and HNF4alpha in regulating pancreas development, beta-cell function and growth. Endocr. Dev. *12*, 33–45.

Marable, S.S., Chung, E., Adam, M., Potter, S.S., and Park, J.S. (2018). Hnf4a deletion in the mouse kidney phenocopies Fanconi renotubular syndrome. JCI Insight *3*, 97497.

Nguyen, T.B., Louie, S.M., Daniele, J.R., Tran, Q., Dillin, A., Zoncu, R., Nomura, D.K., and Olzmann, J.A. (2017). DGAT1-Dependent Lipid Droplet Biogenesis Protects Mitochondrial Function during Starvation-Induced Autophagy. Dev. Cell *42*, 9–21.e5.

Numakura, C., Hashimoto, Y., Daitsu, T., Hayasaka, K., Mitsui, T., and Yorifuji, T. (2015). Two patients with HNF4A-related congenital hyperinsulinism and renal tubular dysfunction: A clinical variation which includes transient hepatic dysfunction. Diabetes Res. Clin. Pract. *108*, e53–e55.

O'Toole, J.F. (2014). Renal manifestations of genetic mitochondrial disease. Int. J. Nephrol. Renovasc. Dis. *7*, 57–67.

Palanker, L., Tennessen, J.M., Lam, G., and Thummel, C.S. (2009). Drosophila HNF4 regulates lipid mobilization and beta-oxidation. Cell Metab. *9*, 228–239.

Palu, R.A., and Thummel, C.S. (2016). Sir2 Acts through Hepatocyte Nuclear Factor 4 to maintain insulin Signaling and Metabolic Homeostasis in Drosophila. PLoS Genet. *12*, e1005978.

Reichold, M., Klootwijk, E.D., Reinders, J., Otto, E.A., Milani, M., Broeker, C., Laing, C., Wiesner, J., Devi, S., Zhou, W., et al. (2018). Glycine Amidinotransferase (GATM), Renal Fanconi Syndrome, and Kidney Failure. J. Am. Soc. Nephrol. *29*, 1849–1858.

Schmittgen, T.D., and Livak, K.J. (2008). Analyzing real-time PCR data by the comparative C(T) method. Nat. Protoc. *3*, 1101–1108.

Shao, X., Somlo, S., and Igarashi, P. (2002). Epithelial-specific Cre/lox recombination in the developing kidney and genitourinary tract. J. Am. Soc. Nephrol. *13*, 1837–1846.

Shizu, R., Osabe, M., Perera, L., Moore, R., Sueyoshi, T., and Negishi, M. (2017). Phosphorylated Nuclear Receptor CAR Forms a Homodimer To Repress Its Constitutive Activity for Ligand Activation. Mol. Cell. Biol. *37*, e00649-16.

Stanescu, D.E., Hughes, N., Kaplan, B., Stanley, C.A., and De León, D.D. (2012). Novel presentations of congenital hyperinsulinism due to mutations in the MODY genes: HNF1A and HNF4A. J. Clin. Endocrinol. Metab. *97*, E2026–E2030.

Sun, K., Montana, V., Chellappa, K., Brelivet, Y., Moras, D., Maeda, Y., Parpura, V., Paschal, B.M., and Sladek, F.M. (2007). Phosphorylation of a conserved serine in the deoxyribonucleic acid binding domain of nuclear receptors alters intracellular localization. Mol. Endocrinol. *21*, 1297–1311.

Thomas, M., Yu, Z., Dadgar, N., Varambally, S., Yu, J., Chinnaiyan, A.M., and Lieberman, A.P. (2005). The unfolded protein response modulates toxicity of the expanded glutamine androgen receptor. J. Biol. Chem. *280*, 21264–21271.

Walsh, S.B., Unwin, R., Kleta, R., Van't Hoff, W., Bass, P., Hussain, K., Ellard, S., and Bockenhauer, D. (2017). Fainting Fanconi syndrome clarified by proxy: a case report. BMC Nephrol. *18*, 230.

Weavers, H., Prieto-Sánchez, S., Grawe, F., Garcia-López, A., Artero, R., Wilsch-Bräuninger, M., Ruiz-Gómez, M., Skaer, H., and Denholm, B. (2009). The insect nephrocyte is a podocyte-like cell with a filtration slit diaphragm. Nature *457*, 322–326.

Wisely, G.B., Miller, A.B., Davis, R.G., Thornquest, A.D., Jr., Johnson, R., Spitzer, T., Sefler, A., Shearer, B., Moore, J.T., Miller, A.B., et al. (2002). Hepatocyte nuclear factor 4 is a transcription factor that constitutively binds fatty acids. Structure *10*, 1225–1234.

Yamagata, K., Furuta, H., Oda, N., Kaisaki, P.J., Menzel, S., Cox, N.J., Fajans, S.S., Signorini, S., Stoffel, M., and Bell, G.I. (1996). Mutations in the hepatocyte nuclear factor-4alpha gene in maturity-onset diabetes of the young (MODY1). Nature *384*, 458–460.

Yu, G., Wang, L.G., Han, Y., and He, Q.Y. (2012). clusterProfiler: an R package for comparing biological themes among gene clusters. OMICS *16*, 284–287.

Zhang, F., Zhao, Y., Chao, Y., Muir, K., and Han, Z. (2013a). Cubilin and amnionless mediate protein reabsorption in Drosophila nephrocytes. J. Am. Soc. Nephrol. *24*, 209–216.

Zhang, F., Zhao, Y., and Han, Z. (2013b). An in vivo functional analysis system for renal gene discovery in Drosophila pericardial nephrocytes. J. Am. Soc. Nephrol. *24*, 191–197.

Zhuang, S., Shao, H., Guo, F., Trimble, R., Pearce, E., and Abmayr, S.M. (2009). Sns and Kirre, the Drosophila orthologs of Nephrin and Neph1, direct adhesion, fusion and formation of a slit diaphragm-like structure in insect nephrocytes. Development *136*, 2335–2344.

STAR★METHODS

KEY RESOURCES TABLE

REAGENT or RESOURCE	SOURCE	IDENTIFIER
Antibodies		
Rat monoclonal anti-HA	Roche	Cat# 11867423001, RRID:AB_390918
Mouse monoclonal anti-ATP5A	Abcam	Cat# ab14748, RRID:AB_301447
Rabbit anti-GFP	Antibody platform Institut Curie	N/A
Mouse monoclonal lamin Dm0	Developmental Studies Hybridoma Bank	Cat# adl84.12, RRID:AB_528338
Mouse Mono- and polyubiquitinylated conjugates monoclonal antibody (FK2)	Enzo Life Sciences	Cat# BML-PW8810, RRID:AB_10541840
Mouse monoclonal anti-PDI (1D3)	Enzo Life Sciences	Cat# ADI-SPA-891, RRID:AB_10615355
Rabbit anti-p62/Ref(2)P	gift from T.E. Rusten	N/A
Mouse monoclonal anti-V5	Invitrogen	Cat# R96025, RRID:AB_159313
Chemicals, Peptides, and Recombinant Proteins		
Paraformaldehyde	Science Services	E15710
Glutaraldehyde	Sigma-Aldrich	G7526
Triton X-100	Roth	3051.3
mounting medium: Roti-Mount FluorCare	Roth	HP19.1
X-gal	ThermoScientific	R0404
polybrene	Santa Cruz	sc-134220
Mayer's Hematoxylin Solution	Sigma-Aldrich	MHS16
Oil Red O solution	Sigma-Aldrich	O1391
BODIPY 493/503	Invitrogen	D3922
Takyon No ROX SYBR 2X MasterMix blue dTTP	Eurogentec	UF-NSMT-B0701
Oligomycin	Santa Cruz	sc-203342
FCCP	Santa Cruz	sc-203578
(+)-Etomoxir sodium salt	Santa Cruz	sc-215009
Antimycin A	Santa Cruz	sc-202467
Rotenone	Santa Cruz	sc-203242
Critical Commercial Assays		
QuikChange II Site-directed Mutagenesis kit	Agilent Technologies	200523
QIAzol Lysis Reagent	QIAGEN	79306
RNeasy Plus Universal Mini Kit	QIAGEN	73404
QuantiTect Reverse Transcription Kit	QIAGEN	205311
Odissey Infrared EMSA Kit	Li-COR	829-07910
Deposited Data		
RNA-seq data at GEO	N/A	#GSE139674
Experimental Models: Cell Lines		
Monkey COS-7 cells	From V. Cantagrel	N/A
HEK293T/17 cells	ATCC	CRL-11268
Mouse embryonic fibroblasts (MEFs) reprogrammed into induced renal epithelial cells (iRECs)	Primary cells	N/A
Experimental Models: Organisms/Strains		
D. melanogaster: Dot-Gal4 driver: w; P[mw, Dot-Gal4]	From Z. Han	N/A
D. melanogaster: Sns-Gal4 driver: w; P[mw, sns-GCN-Gal4]	From S. Abmayr	N/A

(*Continued on next page*)

Continued

REAGENT or RESOURCE	SOURCE	IDENTIFIER
D. melanogaster: RNAi of GFP: w[1118]; P{w[+mC] = UAS-GFP.dsRNA.R}142	Bloomington Drosophila Stock Center	BDSC #9330
D. melanogaster: RNAi of Pkd2: y[1] sc[*] v[1] sev[21]; P{y[+t7.7] v[+t1.8] = TRiP.HMC03263}attP2	Bloomington Drosophila Stock Center	BDSC #51502
D. melanogaster: RNAi of Hnf4: y[1] v[1]; P{y[+t7.7] v[+t1.8] = TRiP.JF02539}attP2	Bloomington Drosophila Stock Center	BDSC #29375
D. melanogaster: RNAi of midway: y[1] sc[*] v[1] sev[21]; P{y[+t7.7] v[+t1.8] = TRiP.HMC06242}attP40/CyO	Bloomington Drosophila Stock Center	BDSC #65963;
D. melanogaster: GFP- and FLAG-tagged Hnf4: w[1118]; PBac{y[+mDint2] w[+mC] = Hnf4-GFP.FLAG}VK00033	Bloomington Drosophila Stock Center	BDSC #38649
D. melanogaster: hsp70-GAL4-dHNF4; UAS-nlacZ56	From Palanker/Thummel	N/A
D. melanogaster: w; P[mw, UAS-GFP-mCherry-Atg8]	From Dr. G. Juhasz	N/A
D. melanogaster: RNAi of Hnf4: w1118; P{UAS-Hnf4-RNAi-GD4362}	Vienna Drosophila Resource Center	VDRC #12692
D. melanogaster: RNAi of ATP6AP2: w; P[mw,UAS-dsRNA-ATP6AP2]KK107676	Vienna Drosophila Resource Center	VDRC #105281;
D. melanogaster: P[mw, UAS-Hnf4-3xHA]	FlyORF	F000144
Oligonucleotides		
Probe: IRD700-AAAGGTCCAAAGGGCGCCT	Metabion	N/A
Other oligonucleotides, see Table S3.	N/A	N/A
Recombinant DNA		
Plasmid: pGW.UAS-HNF4-3xHA.attB	FlyORF	N/A
HNF4 alpha (NM_000457) Human Myc-tagged ORF Clone	Origene	Cat# RC217863
pWPXLd lentiviral vector	Addgene	Cat# 12258
plasmids psPax2	Addgene	Cat# 12260
pMD2.G	Addgene	Cat# 12259
Software and Algorithms		
ImageJ		https://imagej.nih.gov/ij/
Imaris	N/A	https://imaris.oxinst.com
Galaxy platform	Afgan et al., 2016	https://usegalaxy.org
Other		
FastQC version 0.11.5	N/A	http://www.bioinformatics.babraham.ac.uk/projects/fastqc/
Trim Galore! version 0.4.3	N/A	http://www.bioinformatics.babraham.ac.uk/projects/trim_galore/

LEAD CONTACT AND MATERIALS AVAILABILITY

Further information and requests for resources should be directed to and will be fulfilled by the Lead Contact, Matias Simons (matias.simons@institutimagine.org). All plasmids, oligonucleotides, cell lines, and fly strains generated in this study are available with no restriction.

EXPERIMENTAL MODEL AND SUBJECT DETAILS

Fly strains

Drosophila melanogaster stocks were raised on standard cornmeal food. Crosses were reared either at 25°C or at 18°C (e.g., dHNF4^{lowOE}). For experiments performed at 29°C (e.g., dHNF4^{highOE}), crosses were first kept at 25°C or 18°C until mid-to-late second instar before switching to 29°C for a duration of 24h. For the precise genotype used in each figure, see also Table S2.

Cell lines

COS-7 cells (gift by Dr. Cantagrel) were cultured in DMEM (Dulbecco's modified Eagle's medium; GIBCO) supplemented with 10% (v/v) FBS (fetal bovine serum; GIBCO) penicillin/streptomycin and glutamine and were maintained in a humidified atmosphere of 5% CO_2.

iRECs were cultured on 0.1% gelatin-coated flasks in DMEM (Dulbecco's modified Eagle's medium; Lonza) supplemented with 10% (v/v) FBS (fetal bovine serum; GIBCO) penicillin/streptomycin and glutamine and were maintained in a humidified atmosphere of 5% CO_2. iRECs were generated from primary embryonic fibroblasts of male mice (MEFs; see below).

METHOD DETAILS

Generation of fly strains

The following stocks were used: *dot*-Gal4 and *sns*-Gal4 drivers, UAS-*GFP*-RNAi, UAS-*Pkd2*-RNAi, UAS-*dHNF4*-RNAi TRIP, UAS-*midway*-RNAi, *hnf4*-HNF4-GFP.Flag and *hsp70*-GAL4-dHNF4; UAS-nlacZ56 were all obtained from Bloomington Stock Center. UAS-*dHNF4*-RNAi, UAS-*ATP6AP2*-RNAi were obtained from VDRC and UAS-HNF4-3XHA was obtained from FlyORF. UAS-GFP-mCherry-Atg8 was a kind gift from Dr. G. Juhasz. For more info see Key Resources Table.

UAS-dHNF4^{R167W}-, UAS-dHNF4$^{R167W/S169A}$-, UAS-dHNF4^{S169A}- and UAS-dHNF4^{R171W} expressing transgenic flies have been created by site-directed mutagenesis using the QuikChange II Site-directed Mutagenesis kit (Agilent) according to the manufacturer's instructions. Mutagenesis was performed on the pGW.UAS-dHNF4-3xHA.attB plasmid (kindly provided by FlyORF) containing the WT extended gene region of HNF4 using the oligos listed in Table S3. All constructs were injected into flies with the attP landing site at 86FB by Bestgene.

Immunohistochemistry, lipid droplet labeling and tissue imaging

Garland nephrocytes from third instar larvae were dissected in PBS, fixed for 20 min in 4% paraformaldehyde in PBS, washed three times in PBS-T (PBS + 0.1% Triton X-100), and incubated overnight at 4°C with primary antibodies diluted in PBS-T. After washing, tissues were incubated for 2 h at room temperature with secondary antibodies (dilution 1:200) and Hoechst (0.5 μg ml^{-1}) diluted in PBS-T. Tissues were washed twice in PBS-T followed by a wash in PBS. Tissues were mounted in mounting medium Roti®-Mount FluorCare (Roth). Primary antibodies used were rat anti-HA (1:500, Roche), mouse anti-ATP5A (1:100 Abcam), rabbit anti-GFP (1:500, Antibody platform Institut Curie), mouse lamin Dm0 (5 μg/ml, DSHB), mouse anti-mono- and poly-ubiquitinylated conjugates (1:500, Enzo Life Sciences), mouse anti-PDI (1:500, Enzo Life Sciences), rabbit anti-p62/Ref(2)P (1:5000, gift from T.E. Rusten). Secondary antibodies used were fluorescent conjugated Alexa Fluor 488, Alexa Fluor 555, Alexa Fluor 633, and Alexa Fluor 647 (Invitrogen Molecular Probes). For LD labeling, BODIPY 493/503 (2.5 μg ml^{-1}, Molecular Probes) was incubated together with secondary antibodies.

Stainings of pericardial nephrocytes were performed by dissecting the abdomen of 2 days-old female flies in cold PBS. After fixation and washing as mentioned above, tissues were incubated for 2 h at room temperature with Hoechst and horseradish peroxidase (HRP)-Cy3 conjugated (1:100, Jackson Immunoresearch lab) for staining of the cell surface. Tissues were washed and mounted as above.

All images were acquired on a Leica TCS SP8 equipped with a 405-nm laser line and a white light laser with a 63x/1.4 DIC Lambda blue Plan Apochrome objective.

X-gal staining

Vials with hsp70-GAL4-dHNF4; UAS-nlacZ56 third instar larvae were heat-shocked in a water bath at 37°C for 30 minutes and allowed to recover for 6 hours at 25°C. Nephrocytes were then dissected in PBS, fixed in 1% glutaraldehyde (Sigma) in PBS for 20 minutes, washed three times in PBS-T and incubated in a pre-warmed 0.2% X-gal solution for 30 minutes at 37°C in the dark. X-gal solution was obtained by adding 8% of X-gal diluted in N,N-dimethylformamide in a pre-warmed X-gal dilution buffer (3mM $K_3Fe[CN]_6$, 3 mM $K_4Fe[CN]_6$, 0,9 mM $MgCl_2$, 0,1% Tween in PBS). Then samples were washed in PBS and mounted on glass slides in mounting medium. Pictures were taken on a Axioplan Zeiss microscope with a 63x Oil objective (N.A. = 1.4).

Transmission electron microscopy

For transmission electron microscopy, nephrocytes from third instar larvae were fixed with 2.5% gluataraldehyde (Sigma) in 1X PHEM buffer pH7.3 overnight at 4°C. Specimens were first post-fixed with tannic acid 1% in 0.1M cacodylate buffer pH7.2 for 30', then with 1% osmium tetroxide for 1 hr in 0.1M cacodylate buffer pH7.2 at RT, before pre-embedding the specimens in 4% agar type 9, dehydrating in a graded series of ethanol and embedding in Epon. After heat polymerization, thin sections were cut with a Leica Ultramicrotome Ultracut UC7 sections (60 nm) and stained with uranyl acetate and lead citrate. Images were taken with a Tecnai SPIRIT (FEI-Thermofisher Company at 120 kV accelerating voltage with a camera EAGLE 4K x 4K FEI-Thermofisher Company).

Cloning and lentivirus production

For expression in COS-7 cells, mutagenesis was performed using the RC217863 construct (Origene) containing the myc-tagged full-length coding region of human HNF4A2. To this end, the following mutations R85W, R89W, S87A, R85W/S87A were inserted via site-directed mutagenesis (QuikChange II, Stratagene, Santa Clara, CA USA). The oligo pairs used to achieve this are listed in Table S3. The full-length wild-type and mutant cDNA clones were all sequence-verified.

For the lentiviral constructs, mouse transcription factors PAX8 and HNF1b were amplified using gene-specific primers coding sequences and cloned into the pWPXLd lentiviral vector (Addgene). The human transcription factor HNF4A was cloned into a pWPXLd vector containing a V5 epitope tag. For insertion of the R85W mutation into the HNF4A coding sequence, the QuikChange Site-Directed Mutagenesis Kit (Agilent) was applied using the primers listed in Table S3. For lentivirus production, the plasmids pWPXLd, psPax2 and pMD2.G (Addgene) were transfected into HEK293T cells using the calcium phosphate method. After 48h, the supernatant was collected and viruses were precipitated with polyethylene glycol, centrifuged at 6800 rpm for 10 minutes in an Avanti JXN-26 ultracentrifuge (Beckman-Coulter) and stored at −80°C until transduction.

EMSA

The doublestranded oligonucleotide probe containing HNF4A-binding site (−192 to −205 from apoA1 promoter from Metabion) labeled with IRDye 700 was used for HNF4A EMSA analysis. The EMSA reaction (20 μL of final volume) contained 1 μL of IRDye 700-labeled DNA probe (50nM) and 2,5 μg of nuclear extracts from COS7 cells transfected with expression plasmids for HNF4A WT or the mutants. The mixture was completed with 2 μL of 10x Binding Buffer, 2 μL of 25mM DTT/2.5% Tween®20 and 1 μL of Poly (dI-dC)1ug/μL from the Odissey® Infrared EMSA kit (LI-COR). The mixture was incubated for 30 min at RT, fractionated on a 4% native polyacrylamide gel containing 50 mMTris, pH 7.5; 0.38 M glycine; and 2 mM EDTA, and imaged by using a Odyssey ® CLx Imaging System.

iREC generation and immunocytochemistry

MEFs were reprogrammed to iRECs as previously described (Kaminski et al., 2016). The only difference to the previous protocol is that only EMX2 was not included as reprogramming factor to increase proximal tubular fate. In short, Ksp-Cre reporter MEFs (Ksp, kidney specific protein, Cadherin-16) were obtained from limbs of E13.5 mouse embryos and kept in MEF medium (MEFM, containing Dulbeccos's modified Eagle's medium (DMEM), 2 mM L-glutamine, penicillin/streptomycin and 10% fetal bovine serum (FBS). After confluency, cells were split 1:4 and transduced lentivirally with the mouse transcription factors PAX8, HNF1B and human transcription factors HNF4A WT or HNF4A R85W. The following combinations of viruses were used for reprogramming: HNF1B + PAX8 (2TF), 2TF + HNF4A WT and 2TF + HNF4A R85W. Lentiviruses were diluted 1:100 to 1:1000 in MEFM containing 8μg mL^{-1} polybrene (Santa Cruz) and transduced for 12 h on 6 consecutive days. Reprogrammed, GFP-positive cells were sorted 14 days after the last viral transduction using a BD FACSAria™ Fusion flow cytometer (Becton Dickinson).

For immunocytochemistry, cells were washed three times in PBS, fixed for 20 min in 4% paraformaldehyde in PBS, blocked for 10 min in PBS + 3% BSA + 0,1% Tween + 0,1% Triton and incubated overnight at 4°C with primary antibodies diluted in PBS + 3% BSA + 0,1% tween + 0,1% triton. After washing, cells were incubated 2 h at room temperature with secondary antibodies (dilution 1:1000) and Hoechst (0.5 μg ml^{-1}) diluted in PBS + 3% BSA + 0,1% tween + 0,1% triton. Cells were washed three times in PBS and mounted in Roti®-Mount FluorCare (Roth). As primary antibody mouse anti-V5 (1:400, Invitrogen) was used, and as secondary antibody fluorescent conjugated Alexa Fluor 647 (Invitrogen Molecular Probes). For lipid droplet staining, iRECs were washed three times in PBS, fixed for 25 min in 4% paraformaldehyde in PBS, washed three times in PBS and incubated for 1h with Oil Red O solution (Sigma) diluted with distilled water in a 3:2 ratio. Cells were counterstained with Mayer's Hematoxylin Solution for 1 min and washed three times in tap water.

Mitochondrial activity measurements

For measurements of the oxygen consumption rate (OCR) with the Seahorse XF bioanalyzer (Agilent Technologies), iRECs and MEFs were seeded at a density of 20000 cells per well in a collagen coated XFe96 cell culture microplate (Agilent Technologies). 12 hours post-plating cells were balanced for 1 hour in unbuffered XF assay media (Agilent Technologies) supplemented for OCR analysis with 2 mM Glutamine, 10 mM Glucose and 1 mM Sodium Pyruvate. For OCR measurements, compounds were injected during the assay at the following final concentrations: oligomycin (ATP synthase inhibitor to measure respiration associated with cellular ATP production; 1 μM), FCCP (uncoupling agent to measure the maximal respiration capacity; 1 μM), Rotenone and Antimycin A (ETC inhibitors to measure the non-mitochondrial respiration; 1 μM). The data were normalized to protein content measured in each well using BCA assay (Thermo Fisher Scientific) according to the manufacturer's instructions. Seahorse parameters were calculated as followed: last rate measurement before oligomycin injection – minimum rate measurement after oligomycin injection = ATP production; last rate measurement before first injection – non-mitochondrial respiration rate = basal respiration; maximum rate measurement after FCCP injection – non- mitochondrial respiration = maximal respiration. To determine the contribution of fatty acid oxidation to the OCR, etomoxir (CPT1 inhibitor) was injected at a final concentration of 400 μM before the injection of the above-described compounds. Basal respiration of cells treated with vehicle – Basal respiration of cells treated with etomoxir = basal respiration due to fatty acid oxidation; Basal respiration – Basal respiration due to fatty acid oxidation = basal respiration independent of fatty acid oxidation. For etomoxir experiments results were normalized by cell area measured with IncuCyte® S3 Live-Cell Analysis System.

RNA sequencing and qPCR

Total RNA was extracted with QIAzol Lysis Reagent (QIAGEN) and isolated using the RNeasy Plus Universal Mini Kit (QIAGEN). Library preparation and RNA sequencing were conducted by GATC Biotech AG on an Illumina platform with single-end 50 bp mRNA sequencing. Sequencing data were uploaded to the Galaxy platform (Afgan et al., 2016), and usegalaxy.org server was

used to analyze the data. Quality check was assessed with FastQC version 0.11.5 (http://www.bioinformatics.babraham.ac.uk/projects/fastqc/). Reads were trimmed with Trim Galore! version 0.4.3 (http://www.bioinformatics.babraham.ac.uk/projects/trim_galore/) and aligned to the genome build GRCm38 using RNA STAR2 version 2.5.2b. Average read count after quality control was 64 million. Gene counts were calculated with featureCounts version 1.6.0 and differentially expressed genes (DEG) between MEF and iREC WT and iREC R85W conditions were determined using DESeq2 version 1.18.1.

For quantitative PCR total RNA was extracted from iRECs which were re-sorted using a strict gating of GFP-positive cells. RNA extraction was performed as described above. For reverse transcription to cDNA the QuantiTect Rev. Transcription Kit (QIAGEN) was applied according to manufacturer's instructions. The qPCR was performed on a Roche LightCycler® 480 instrument using 10 ng of cDNA, gene-specific primers and Takyon SYBR® Master Mix (Eurogentec, Takyon No Rox SYBR® MasterMix dTTP Blue, UF-NSMT-B0701). The primers used are listed in Table S3.

qPCR data were analyzed applying the comparative CT method (Schmittgen and Livak, 2008). RNA-seq raw data files have been deposited in GEO (Gene expression omnibus).

QUANTIFICATION AND STATISTICAL ANALYSIS

Image analysis

For nephrocytes, percentage of total cell area occupied by LDs was quantified with the "Analyze Particles"-tool of Fiji on thresholded Z stack projections images of BODIPY 493/503. Average nuclear intensity of dHNF4-GFP staining in nephrocytes and whole ATP5A staining intensity in nephrocytes were measured on the nuclear z-plane by the mean intensity using the "Analyze Measure"- tool of Fiji. LD size and density were measured with Imaris by defining the volume of the cell in 3D and then using the Spot Detector tool. For calculating LD density, the number of LDs was normalized to the total volume of the segmented cell.

For IRECs, the average nuclear intensity of HNF4A-V5 was measured on the nuclear z-plane by the mean intensity using the "Analyze Measure"-tool of Fiji. For LD staining, the percentage of the area occupied by LDs in iRECs was quantified using the "Analyze Particles"-tool of Fiji on thresholded images.

Statistics

All experiments were repeated at least 3 times with consistent results. Statistical analysis was performed with 2-tailed, unpaired Mann-Whitney's t test for comparison of 2 groups, or one-way ANOVA followed by Tukey's or Dunn's multiple comparison test for multiple comparisons after verifying normality. Statistical analyses were performed using the GraphPad Prism 6.0 program. *P* less than 0.05 was considered significant (*$p < 0.05$, **$p < 0.01$, ***$p < 0.001$, ****$p < 0.0001$). More details on statistics can be found in each figure legend.

RNA-seq data analysis

Downstream analysis was performed using R version 3.5.0. A heatmap of the significant DEG (N = 10963) between all three analyzed conditions was drawn using gplots version 3.0.1 (Figure 7E). For this, FPKM (Fragments Per Kilobase Million) values were calculated for every gene, base 10 logarithm was computed and z-score standardization was performed. Significant DEG were then divided into 12 clusters using soft clustering tool Mfuzz (Kumar and Futschik, 2007). Computed clusters were assigned to eight logically expected expression change patterns (Figure S7). We were not able to assign any genes to logically expected cluster number six representing genes being downregulated in iREC WT and mutation conditions compared to MEFs.

A GO over-representation test was done using Bioconductor package clusterProfiler version 3.8.1 (Yu et al., 2012) and not more than five most significant terms from each biological domain of clusters one, three, four, seven and eight were plotted (Figure 7F). With genes representing mitochondrial gene expression GO term (GO:0140053) a heatmap was plotted as outlined above.

DATA AND CODE AVAILABILITY

RNA-seq datasets have been deposited in GEO (Gene expression omnibus) under the accession number #GSE139674.

Cell Reports, Volume *29*

Supplemental Information

Molecular Basis for Autosomal-Dominant Renal Fanconi Syndrome Caused by *HNF4A*

Valentina Marchesin, Albert Pérez-Martí, Gwenn Le Meur, Roman Pichler, Kelli Grand, Enriko D. Klootwijk, Anne Kesselheim, Robert Kleta, Soeren Lienkamp, and Matias Simons

Table S1: Table summarizing the dominant negative effects (genomic and non-genomic) observed in all the different genotypes. Related to Figure 6.

	Genomic effect			Non-genomic effect			
**PHENOTYPE / GENOTYPE**	**Lipid droplets**	**Mito-chondria ATP5A levels**	**Nuclear localization**	**Autophagy**	**ER**	**Animal viability (with *Dot-*GAL4)**	**Nephrocyte Viability (with *Sns-*GAL4)**
**dHNF4 RNAi**	High	Low	/	Normal	Normal	Normal	Normal (not shown)
**dHNF4^{lowOE} (18°C)**	Very low	Normal (data not shown)	Normal	/	Normal	Normal	Normal (not shown)
**dHNF4^{OE} (25°C)**	Dual pheno-type	High (LD-) Normal (LD+)	Normal (LD-) Peripheral (LD+)	High	Normal (LD-) Expanded (LD+)	Normal (loss of NPs in adults)	Reduced size and number
**dHNF4^{highOE} (29°C)**	Very high	Low	Very peripheral or absent	High (not shown)	Very expanded	Early lethality	/
**dHNF4^{S169A} (25°C/29°C)**	Very low	Very high	Normal	Normal	Normal	Normal	Normal (not shown)
**dHNF4$^{R171W/MODY}$ (25°C)**	Low	High	Normal	Normal	Normal	Normal	Normal
**dHNF4$^{R171W/MODY}$ (29°C)**	Low	/	Normal	/	Normal	Normal	/
**dHNF4$^{R167W/FRTS}$ (25°C)**	Very high	Low	Very peripheral or absent	High	Very expanded	Early lethality	Loss of NPs
**dHNF4$^{R167W/FRTS}$ (18°C)**	Very high	Low (not shown)	Very peripheral or absent	/	Very expanded	Early lethality	/
**dHNF4$^{R167W/S169A}$ (25°C)**	High (partial rescue)	Normal	Normal	Normal	Normal	Normal	Normal

/: data not available

Table S2. ***Drosophila*** **genotypes used in this study. Related to STAR Methods.**

Genotype	Figure
w; +/+; *hnf4*-HNF4-GFP.Flag	Figure 1
w; +/+; *hsp70*-GAL4-dHNF4, UAS-nlacZ56	Figure 1
w; *Dot*-Gal4/+; UAS-*GFP-RNAi* (bloo#9330)/+	Figures 1, 2, 4, 6, S1, S2, S3, S4, S5, S6
w; *Dot*-Gal4/UAS-*Pkd2-RNAi* (bloo#51502); +/+	Figure S1
w/yv; *Dot*-Gal4/+; UAS-*HNF4-RNAi* (bloo#29375)/+	Figures 1, S1, S4
w; *Dot*-Gal4/UAS-*HNF4-RNAi* (Vienna GD12692); +/+	Figure S1
w; *Dot*-Gal4/+; UAS-HNF4-3xHA/+ (FlyORF #F000144)	Figures 2, 3, S2, S3, S4
w; *Dot*-Gal4/UAS-*midway-RNAi* (bloo#65963); +/+	Figure S2
w/yv; *Dot*-Gal4/+; UAS-*GFP-RNAi*/UAS-*HNF4-RNAi* (bloo#29375)	Figure S2
w/yv; *Dot*-Gal4/UAS- *midway-RNAi*; UAS-*HNF4-RNAi* (bloo#29375)/+	Figure S2
w; *Dot*-Gal4/UAS-*midway-RNAi*; UAS-HNF4-3xHA/+	Figure S2
w; *Dot*-Gal4/+; UAS-HNF4^{R167W}-3xHA/+	Figures 5, 6, S6
w; *Dot*-Gal4/+; UAS-HNF4^{R171W}-3xHA/+	Figures 5, 6, S6
w; *Dot*-Gal4/+; UAS-HNF4^{S169A}-3xHA/+	Figures 4, S5
w; *Dot*-Gal4/+; UAS-HNF4$^{R167W/S169A}$-3xHA/+	Figures 5, 6
w; *Dot*-Gal4/ UAS-*midway-RNAi*; UAS-HNF4^{R167W}-3xHA/+	Figure S6
w; *Dot-Gal4*/UAS-*Pkd2-RNAi*; *hnf4*-HNF4-GFP.Flag/+	Figure 4
w; *Dot-Gal4*/+; *hnf4*-HNF4-GFP.Flag/UAS-HNF4-3xHA	Figure 4
w; *Dot-Gal4*/+; *hnf4*-HNF4-GFP.Flag/UAS-HNF4^{R167W}-3xHA	Figure 5
w; *Dot-Gal4*/+; *hnf4*-HNF4-GFP.Flag/UAS-HNF4^{R171W}-3xHA	Figure 5
w; *Dot-Gal4*/+; *hnf4*-HNF4-GFP.Flag/UAS-HNF4^{S169A}-3xHA	Figure 4
w; *Dot-Gal4*/+; *hnf4*-HNF4-GFP.Flag/UAS-HNF4$^{R167W/S169A}$-3xHA	Figure 5
Dot-Gal4; UAS-GFP-mCherry-Atg8/UAS-*Pkd2-RNAi*; +/+	Figures S4, S5, S6
Dot-Gal4; UAS-GFP-mCherry-Atg8/UAS-*ATP6AP2-RNAi* (Vienna KK105281); +/+	Figure S4
w; *Dot-Gal4/*UAS-GFP-mCherry-Atg8; UAS-HNF4-3XHA/+	Figure S4
w; *Dot-Gal4/*UAS-GFP-mCherry-Atg8; UAS-HNF4^{R167W}-3XHA/+	Figure S6

Dot-Gal4; UAS-GFP-mCherry-Atg8/+; UAS-HNF4^{R171W}-3XHA/+	Figure S6
w; *Dot-Gal4/*UAS-GFP-mCherry-Atg8; UAS-HNF4$^{R167W/S169A}$-3XHA/+	Figure S6
Dot-Gal4; UAS-GFP-mCherry-Atg8/+; UAS-HNF4^{S169A}-3XHA/+	Figure S6
w; *Sns*-GAL4/+; UAS-*GFP-RNAi*/+	Figures 6, S1, S4
w; *Sns*-GAL4/+; UAS-*HNF4-RNAi* (bloo#29375)/+	Figure S1
w; *Sns*-GAL4/+; UAS-HNF4-3xHA/+	Figure S4
w; *Sns*-GAL4/+; UAS-HNF4^{R167W}-3xHA/+	Figure 6
w; *Sns*-GAL4/+; UAS-HNF4^{R171W}-3xHA/+	Figure 6
w; *Sns*-GAL4/+; UAS-HNF4$^{S169A/R167W}$-3xHA/+	Figure 6

Table S3. Oligonucleotide information. Related to STAR Methods.

Samples	Oligonucleotides
Mutagenesis in flies: UAS-HNF4^{R167W}	forward:CATTCTTCTGGAGGAGTGTCAGGAAAAATCATCAG<br>reverse:ACTCCTCCAGAAGAATCCTTTGCAGCCGTCGCAGC
Mutagenesis in flies: UAS-HNF4^{S169A}	forward:CAGGAGGGCTGTCAGGAAAAATCATCAGTACAC<br>reverse:CCTGACAGCCCTCCTGAAGAATCCTTTGCAGC
Mutagenesis in flies: UAS-HNF4$^{R167W/S169A}$	forward:CTGGAGGGCTGTCAGGAAAAATCATCAGTACAC<br>reverse:CCTGACAGCCCTCCAGAAGAATCCTTTGCAGC
Mutagenesis in flies: UAS-HNF4^{R171W}	forward:GGAGTGTCTGGAAAAATCATCAGTACACTTGCAG<br>reverse:GGAGTGTCTGGAAAAATCATCAGTACACTTGCAG
Mutagenesis in COS-7 cells: R85W	forward:CGCACGCTCCTCCAGAAGAAGCCCTTG<br>reverse:CAAGGGCTTCTTCTGGAGGAGCGTGCG
Mutagenesis in COS-7 cells: R89W	forward:CATGTGGTTCTTCCACACGCTCCTCCGGA<br>reverse:TCCGGAGGAGCGTGTGGAAGAACCACATG
Mutagenesis in COS-7 cells: S87A	forward:GGTTCTTCCGCACGGCCCTCCGGAAGAAGC<br>reverse:GCTTCTTCCGGAGGGCCGTGCGGAAGAACC
Mutagenesis in COS-7 cells: R85W/S87A	forward:GGTTCTTCCGCACGGCCCTCCAGAAGAAGCCCTTGC<br>reverse:GCAAGGGCTTCTTCTGGAGGGCCGTGCGGAAGAACC
Mutagenesis on human HNF4A: R85W	forward:GGGCTTCTTCTGGAGGAGCGT<br>reverse:GTCGACACTGCCGACGTT
qPCR primers: Tbp	forward:CCCCTTGTACCCTTCACCAAT<br>reverse:GAAGCTGCGGTACAATTCCAG
qPCR primers: HNF4A	forward:GGTGTCCATACGCATCCTTGAC<br>reverse:AGCCGCTTGATCTTCCCTGGAT
qPCR primers: V5.HNF4A	forward:GTAAGCCTATCCCTAACCCTCTC<br>reverse:AAATTCCAGGGTGGTGTAGG
